# Targeting MED23 inhibits hepatocellular carcinoma development by suppressing compensatory proliferation and facilitating ROS-mediated cell death

**DOI:** 10.1038/s41419-025-08348-8

**Published:** 2025-12-24

**Authors:** Xiaying Zhao, Zhichao Wang, Lihua Min, Dan Cao, Yajing Chu, Chonghui Li, Jiabin Cai, Gang Wang

**Affiliations:** 1 State Key Laboratory of Genetics and Development of Complex Phenotypes, School of Life Sciences and Zhongshan Hospital, Fudan University, Shanghai, 200438 China; 2 State Key Laboratory of Cell Biology, CAS Center for Excellence in Molecular Cell Science, Shanghai Institute of Biochemistry and Cell Biology, Chinese Academy of Sciences, University of Chinese Academy of Sciences, Shanghai, 200031 China; 3 Department of Liver Surgery and Transplantation, Key Laboratory of Carcinogenesis and Cancer Invasion, Shanghai Key Laboratory of Organ Transplantation, Liver Cancer Institute, Zhongshan Hospital, Fudan University, Shanghai, 200032 China

**Keywords:** Oncogenes, Biochemistry

## Abstract

Hepatocellular carcinoma (HCC) is frequently linked to compensatory proliferating hepatocytes in damaged livers, yet the underlying molecular mechanisms remain elusive. The Mediator complex precisely coordinates multiple transcription factors and cofactors to regulate diverse physiological and pathological processes. Here, we discovered that Mediator subunit MED23 is involved in the progression of HCC. Both constitutive and inducible liver-specific ablation of Med23 effectively inhibited HCC development in diethylnitrosamine (DEN)-induced HCC mouse models. Mechanistically, MED23 deficiency significantly compromised hepatocyte cell viability by reducing the stability of the NQO1 protein, thereby leading to an increase in reactive oxygen species (ROS) production. Furthermore, MED23 collaborates with the transcription factor RFX5 to regulate a novel enhancer function for IGF2 expression, which thus influences hepatocyte viability and HCC development. Consistently, overexpression of IGF2 in MED23-deficient HCC cells stabilizes NQO1 and partially restores cell growth and reduces apoptosis. Collectively, our findings underscore the significance of the MED23-IGF2-NQO1 axis in HCC progression and propose a novel therapeutic strategy for the treatment of HCC.

## Introduction

Hepatocellular carcinoma (HCC), the major type of primary liver cancer, is one of the most common cancers and the second leading cause of cancer related mortality worldwide [1, 2]. Major HCC risk factors include aflatoxin, infection with hepatitis B virus (HBV) or hepatitis C virus (HCV), and cirrhosis associated with chronic liver injury [3]. Additionally, metabolic syndrome associated with obesity and non-alcoholic fatty liver disease (NAFLD) is becoming a predominant factor due to lifestyle changes [4–7]. Although great progress has been made in the treatment of HCC, such as the multi-kinase inhibitors sorafenib and lenvatinib, the 5-year survival rate for advanced HCC patients remains low [1, 3, 8]. Therefore, it is urgent to identify novel drug targets and to elucidate the underlying molecular mechanisms of hepatocarcinogenesis to provide new therapeutic strategies for HCC patients.

In order to better understand the pathogenesis of HCC, many mouse models have been employed. The most widely used mouse model is the diethylnitrosamine (DEN)-induced HCC model, which recapitulates human disease characterized by chronic liver injury and inflammation followed by tumor formation [9, 10]. As a DNA-adduct-formation agent, a single injection of the carcinogen DEN on postnatal day 14 induces reactive oxygen species (ROS) accumulation and DNA damage within liver parenchymal cells, which subsequently leads to cell death [11]. Then Kupffer cells, the resident hepatic macrophages, are activated by the factors secreted from damaged hepatocytes. Activated Kupffer cells can secrete abundant pro-inflammatory and pro-proliferative cytokines, thereby driving the compensatory proliferation of surviving hepatocytes, triggering the formation of initiated liver cancer cells, which further propagates into neoplastic foci promoted by multiple rounds of cell death–inflammation–regeneration [12, 13]. Thus, the ROS-induced cell death and subsequent compensatory proliferation are essential for liver tumor initiation.

NAD(P)H:quinone oxidoreductase 1 (NQO1) is an enzyme that is significantly upregulated in human liver cancer and plays a crucial role in the antioxidant defense system as well as apoptosis evasion [14–17]. Upon accumulation of ROS, Nuclear factor erythroid 2- related factor 2 (NRF2) is stabilized and activates NQO1 and other protective enzymes transcriptionally. Noticeably, NQO1 protein level was dramatically increased in tumors, but its mRNA level was just modestly altered compared to non-tumors [14]. Given the importance of cellular homeostasis, we hypothesized that the translation and/or protein stability of NQO1, rather than its transcription, could be tightly controlled and disruption of this process likely affects the carcinogenesis.

The insulin-like growth factors (IGFs), including IGF1 and IGF2, regulate cell growth and differentiation in many species. IGF1 acts as a major determinant of somatic growth, largely dependent on growth hormone (GH) signaling [18]. *IGF2*, perhaps the most intricately regulated of all growth factors, is imprinted, or expressed monoallelically, and active only on the paternally inherited allele both in mouse and human. Imprinted *IGF2* occurs in clusters with the oppositely maternally imprinted, non-coding gene *H19* [19]. Interestingly, *IGF2* and *H19* genes share serial distal enhancers that act on either gene, depending on parental origin. The precise regulation of *IGF2* highlights its importance in development and disease. Sustained IGF2 action commonly promotes carcinogenesis and is associated with a poor prognosis [20, 21], including in liver cancer [22, 23]. One study reported that IGF2 was upregulated in a large proportion of HCC samples through demethylation of its promoter, and an antibody against IGF1 and IGF2 could impair the growth of xenograft tumors and increase the survival of these mice [24]. These studies highlight the key role of IGF2 in liver cancer; however, the precise mechanisms of transcriptional regulation of IGF2, especially the potential upstream transcriptional regulatory factors, as well as downstream effectors of IGF2/IGF1R signaling are largely uncharacterized.

Mediator complex is an evolutionarily conserved, multi-subunit protein complex that is best known to connect transcription factors (TFs) with RNA polymerase II (Pol II) machinery for precise transcription output [25–29]. Emerging evidence implicates Mediator subunits such as MED1, MED12, and CDK8 in cancer progression and metabolism [30–32]. We previously showed that Mediator subunit MED23 plays critical roles in multiple cell fate determination and cancer development [33, 34]. Through constructing liver-specific Med23 deletion model, we revealed that hepatic MED23 regulates glucose and lipid metabolism as well as carbon tetrachloride (CCl_4_)-induced liver fibrosis [35, 36]. These findings prompted us to investigate the function of MED23 in HCC development. In this study, we analyzed the critical role of MED23 in the HCC mouse models, HCC cell lines, and human clinical samples, and we have identified a MED23-IGF2-NQO1 axis in HCC development through mechanisms of inhibiting the liver cell death while enhancing the compensatory proliferation. Our findings suggest that Mediator MED23 emerges as a key player in hepatocarcinogenesis and may serve as a potential therapeutic target.

## Methods

### Animals and treatment

*Med23*-floxed (*med23*^f/f^) mice were generated using homologous recombination [34]. Male mice were used in this study and were maintained on a mixed genetic background (C57BL/6; 129 Sv). The constitutive and inducible deletion of *Med23* in livers was performed as previously described [35, 37]. Briefly, *Mx-Cre*; *med23*^f/f^ mice (*med23*^Δli^*) were injected intraperitoneally with poly(I:C) (13 mg/kg body weight) to delete the floxed alleles in the liver. To induce HCC formation in mice, 14-day-old male mice were treated with a single intraperitoneal injection of DEN (Sigma-Aldrich, 50 mg/kg body weight). Then, mice were euthanized and analyzed at 9 months of age. All animals were maintained in grouped cages in a temperature-controlled, pathogen-free facility on a 12/12-h light/dark cycle and fed with a standard chow diet. Liver tissues were collected for histological, biochemical, and molecular analyses as described below.

### Histological analysis

H&E and immunohistochemistry in formalin-fixed paraffin-embedded sections were performed as described previously [37, 38]. The following antibodies were used for staining: anti-Ki67 (Novocastra, NCL-Ki67p), anti-pH3S10 (Upstate, 06-570), anti-PCNA (Neomarker, Ms-106-p0) and anti-8OH-dG (Abcam, ab62623).

TdT-mediated dUTP nick-end labeling (TUNEL) assays were performed using the Apoptosis DNA Fragmentation Assay Kit (Clontech, #630107) to detect apoptotic cells. To detect proliferating cells, mice were injected intraperitoneally with 1 mg/kg BrdU (Sigma-Aldrich). Incorporated BrdU was detected in formalin-fixed paraffin-embedded sections using mouse monoclonal α-BrdU antibody (Sigma-Aldrich, B2531). A minimum of five different fields in each liver section were used to count the number of signal-positive cells.

### Biochemical parameters of serum

Collected blood was allowed to clot for 1 h at 4 °C and centrifuged at 3000 × *g* for 10 min twice. Isolated supernatant serum was used to measure total bilirubin, alanine aminotransferase (ALT) and aspartate aminotransferase (AST) activity according to the manufacturer’s instructions (Shensuoyoufu, Shanghai, China).

### Primary hepatocyte culture

Primary hepatocytes were isolated from 8 to 12 week old mice by collagenase perfusion as described previously [39]. Hepatocytes were plated on 6-well plates and cultured in Dulbecco’s modified Eagle medium supplemented with 10% fetal bovine serum (Hyclone). The cells were washed with PBS and harvested 24 h later.

### Cell culture and plasmids

HCC cell lines HepG2, Huh7, Tong, Hep3B, and Huh6 were gifted from Lijian Hui (Institute of Biochemistry and Cell Biology, Shanghai). They were cultured in Dulbecco’s modified Eagle medium supplemented with 10% fetal bovine serum (Hyclone). All cells were incubated at 37 °C in 5% CO_2_ humidified air. Stable *MED23* knockdown HCC cell lines were established according to the manufacturer’s recommendation (Clontech), and were previously described [33, 40]. Briefly, retroviruses were produced by cotransfecting recombinant pSiren-RetroQ vector with pCL10A1 helper plasmid into 293T cells using Lipofectamine 2000 (Invitrogen). Infected cells were selected with puromycin (Sigma-Aldrich) after spin infection (1258 × *g*, 30 °C, 90 min).

### Cell viability assay

The growth ability of cells was assessed by the CellTiter-Glo® Luminescent Cell Viability Assay (Promega). Stable shCtrl and shMED23 cells were trypsinized and equal numbers of cells were seeded in 96-well plates. Cells were allowed to grow for an additional 7 days. Cell numbers were measured daily according to the manufacturer’s recommendation (Promega).

### Flow cytometric apoptosis analysis

ShCtrl and shMED23 cells were seeded in 6-cm dish after puromycin selection. Then the cells were harvested, washed in PBS followed by staining with Annexin V and PI for 30 min (Invitrogen) when cell density reached 80%. The samples were then recorded using Flow Cytometer.

### siRNA transfection

Tong cells were transfected with the indicated siRNAs (10 μM, obtained from GenePharma, Shanghai, China) using RNAiMax transfection reagent (Invitrogen, 13778150) according to the manufacturer’s instructions. At 48 h after transfection, cells were collected for protein and RNA extraction. The corresponding siRNA sequences are detailed in Supplementary Table 1.

### Protein and RNA analysis

Proteins were extracted from mouse tissues and HCC cell lines as previously described [36]. Briefly, fresh mouse tissues were excised and frozen in liquid nitrogen at once. Then tissues were homogenized in RIPA lysis buffer (Beyotime, P0013B) added with complete protease inhibitor cocktail (Roche, 4693116001). Equal amount of total protein of tissue lysates from each condition were resolved by 8–12% SDS-PAGE followed by immunoblotting. HRP-conjugated secondary antibodies were purchased from the Jackson Laboratory. The following primary antibodies were used for western blotting: anti-MED23 (Abcam, ab200351), anti-IGF2 (Abcam, ab124964), anti-IGF1R (Proteintech, 20254-1-AP), anti-pIGF1R (Cell Signaling Technology, #6113), anti-γ-H2AX (Cell Signaling Technology, #9718), anti-NQO1 (Abcam, ab34173), anti-RFX5 antibody (Proteintech, 12137-1-AP), anti-MED6 antibody (Santa Cruz, sc-9434), anti-CDK8 antibody (BD Biosciences, 552053), anti-FLAG antibody (Sigma-Aldrich, F3165), anti-β-ACTIN (Proteintech, 66009-1-Ig), anti-γ-Tubulin (Sigma-Aldrich, T6557) and anti-GAPDH (Proteintech, 60004-1-Ig).Total RNA was extracted from cells or liver tissues using TRIzol (Thermo, 15596018). 2 μg of total RNA was used for first-strand cDNA synthesis according to the manufacturer’s instructions (Takara), and real-time PCR (RT-PCR) was performed using SYBR Premix Ex Taq^TM^ (Takara) in ABI QuantStudio 6 Realtime PCR machine. All values were normalized to the level of *β-actin* mRNA. The primers were listed in Supplementary Tables 2, 3.

### RNA-Seq and data analysis

CapitalBio Technology (Beijing, China) performed libraries construction and sequencing. The purity and integrity of the extracted RNA were confirmed using an Agilent Bioanalyzer. Libraries were prepared from 150 ng total RNA (TruSeq v2, Illumina), and pair-ended sequencing was performed on an Illumina HiSeq 2500 using bar-coded multiplexing and a 150-bp read length, yielding a median of 34.1 M reads per sample. Raw reads were mapped to mouse reference genome (mm9 assembly) by TopHat with default parameters. Uniquely mapped reads were filtered for downstream analysis, which included ∼80% of raw reads. deSeq was applied to count the number of reads that located on the exons of gene, which was normalized to RPKM as the measurement of mRNA abundance.

### Dual-luciferase reporter assay

The dual-luciferase reporter assay was performed as described [36]. Briefly, Tong cells were seeded into a 12-well plate at 1 × 10^5^ cells per well overnight. These cells were then transfected with a luciferase reporter plasmid and EGFP plasmid along with various expression constructs, as indicated, by Lipofectamine 2000 (Invitrogen, 11668019). All wells were supplemented with control empty expression vector plasmids to keep the total amount of DNA constant. The cells were harvested and subjected to dual-luciferase reporter assays after 24–36 h of transfection according to the manufacturer’s protocol (Promega). For Fig. 7E, Tong cells were first transfected with the control siRNA or RFX5 siRNA (10 μM) using RNAiMax transfection reagent. The next day, these cells were seeded into a 12-well plate for luciferase assay.

### Co-immunoprecipitation (Co-IP) assay

For transient co-transfection, 4 μg Flag-tagged plasmid (FLAG or FLAG-RFX5) and 8 μg Myc-tagged plasmid (MED23-MYC) were cotransfected into 293T cells plated in 10-cm dishes with Lipofectamine 2000 (Invitrogen). After 36 h, the cells were washed with PBS and lysed in 1 mL lysis buffer (1% NP-40, 10% glycerol, 135 mM NaCl, 20 mM Tris, pH 8.0, 10 mM NaF, 2 mM NaVO4, and freshly added protease inhibitors from Roche). After a rotation of 90 min at 4 °C, the lysates were spun down at 13,200 rpm for 15 min. The supernatants were then added to 15 μl of anti-Flag beads (Biolinkedin) and incubated at 4 °C overnight. The beads were washed 3 times with lysis buffer, and boiled with SDS loading buffer. The bounded proteins were analyzed by Western blot with the indicated antibodies.

As for endogenous Co-IP, 293T cells (WT and HA-tag knockin) in 10-cm dishes were lysed in 1 mL PBS buffer containing 5 mM EDTA, 1% Triton X-100 and freshly supplemented with protease inhibitors from Roche. After a rotation of 60 min at 4 °C, the lysates were centrifuged at 13,200 rpm for 15 min. The supernatant was incubated with 15 μl of anti-HA beads (Thermo Scientific, 88837) at 4 °C overnight. The beads were then washed with lysis buffer three times and boiled with SDS loading buffer for Western blot assay with the indicated antibodies.

### Chromatin immunoprecipitation (ChIP)

Tong cells (shCtrl and shMED23) were cross-linked with a final concentration of 1% formaldehyde in culture medium on a shaker for 9 min at room temperature and neutralized by the addition of glycine to a final concentration of 0.125 M for 5 min. After washing with cold PBS, the cells were collected by centrifugation. The cell pellet was resuspended with ChIP lysis buffer (50 mM Tris-HCl, pH 7.4; 1% SDS; and 10 mM EDTA), followed by sonication. The following procedures were performed as described previously [35]. The antibodies used for ChIP were as follows: anti-H3K4me1 (Abcam, ab8895) and anti-H3K27ac (Abcam, ab4729). DNA extracted from ChIP products was analyzed by qRT-PCR with TB Green Premix Ex Taq (Tli RNaseH Plus) (Takara, RR420A). The primers are listed in Supplementary Table 4.

ChIP-seq datasets of HepG2 database (α-RFX5, α-H3K27ac, α-HNF4α, and α-FOXA1) were obtained from the Encyclopedia of DNA Elements (ENCODE) project. Analysis and visualization of data were carried out in IGV software. The survival analysis in TCGA LIHC cohort was determined by Kaplan–Meier plotter browser (https://kmplot.com/analysis/index).

### Statistical analysis

All data are presented as mean ± SEM. Statistical significance was determined using the unpaired Student’s *t* test or Mann–Whitney test, depending on data distribution assessed by the Shapiro–Wilk test. The differences were considered significant when *P* value was <0.05. Statistical calculation was performed using the GraphPad Prism 6 software.

## Results

### MED23 is upregulated in human HCC tumors and is essential for HCC cell growth in vitro

**Fig. 1:**
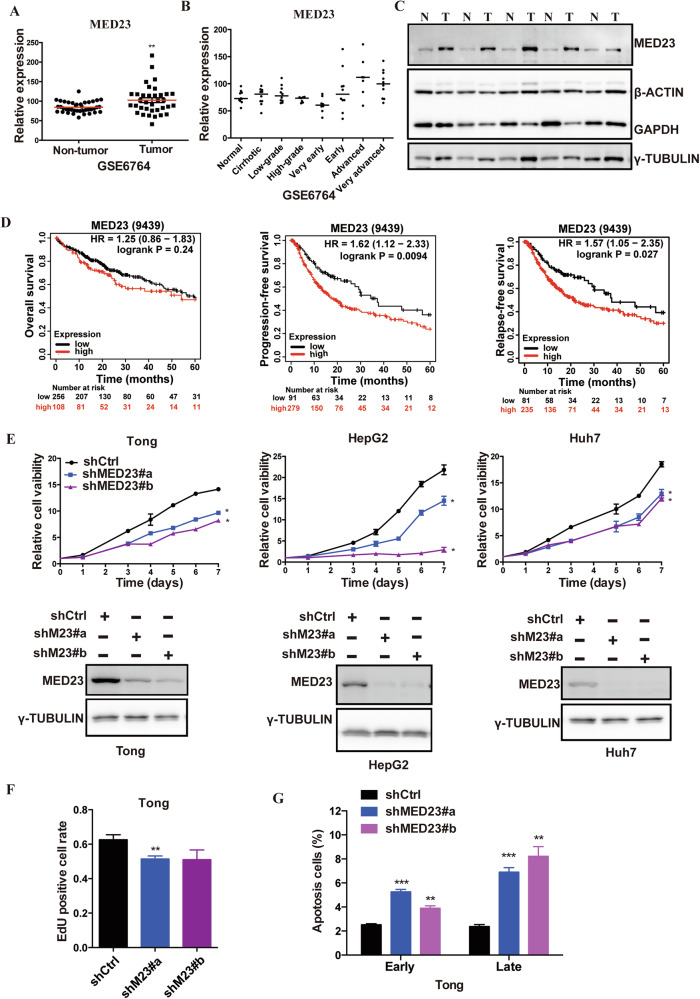
MED23 is upregulated in human HCC tumors and is required for HCC cell viability. **A** Relative *MED23* expression levels in human HCC tumors and adjacent non-tumor samples from the public dataset GSE6764 (Non-tumor, *n* = 40; Tumor, *n* = 35). **B** *MED23* mRNA expression levels in non-tumor samples, cirrhotic, and HCC tumor with different stages from the public GSE6764 dataset. **C** Immunoblot analysis of MED23, γ-TUBULIN, β-ACTIN and GAPDH in tumor (T) and non-tumor (N) liver tissues from human HCC patients. **D** Predicting the 5-year overall survival, progression-free survival, and relapse-free survival of HCC patients according to the expression level of *MED23* (https://kmplot.com). **E** Cell viability analysis of Tong, HepG2, and Huh7 cells transduced with retroviral shCtrl or shMED23 (*n* = 3–6 per group) (Top). Immunoblot analysis of MED23 and γ-tubulin in Tong, HepG2, and Huh7 cells transduced with retroviral shCtrl or shMED23 (Bottom). **F**, **G** See as per msp Tong cells transduced with retroviral shCtrl or shMED23 were subjected to EdU staining (**F**) (*n* = 6 per group) and apoptosis analysis by PI/AnnexinV staining (**G**) (*n* = 3 per group). Data are presented as mean ± SEM. The Shapiro–Wilk test was applied to test the data for normality. Statistical significance was determined using unpaired Student’s *t* test. *P < 0.05, **P < 0.01, ***P < 0.001.

We first asked whether MED23 is dysregulated in human HCC tumors by examining the expression of *MED23* in published HCC datasets. *MED23* mRNA levels were significantly increased in human HCC tumors compared to the adjacent tissues (Fig. 1A; Supplementary Fig. 1A), and increased *MED23* expression correlated with the progression of HCC (Fig. 1B). The protein levels of MED23 were also dramatically augmented in HCC samples compared to paired non-tumor tissues (Fig. 1C). Kaplan–Meier analysis of The Cancer Genome Atlas (TCGA) database demonstrated that high *MED23* expression was associated with a trend toward poorer overall survival in HCC patients (Fig. 1D), and patients with high *MED23* mRNA expression may have a shorter progression-free survival and relapse-free survival time compared to those patients with a low *MED23* (Fig. 1D). These analyses underscore the significance of MED23 in human HCC.

To investigate the cellular function of MED23, we knocked down *MED23* in human HCC cell lines using retrovirus-mediated shRNA, and verified knockdown efficiency by Western blot (Fig. 1E; Supplementary Table 5). *MED23* silencing significantly impaired cell viability (Fig. 1E) and proliferation (Fig. 1F; Supplementary Fig. 1B) in HCC cells. Flow cytometry further indicated that both early and late apoptotic cells increased after *MED23* knockdown (Fig. 1G; Supplementary Fig. 1C). These data suggest that high MED23 expression supports HCC development possibly by suppressing apoptosis and promoting proliferation.

### Med23 is required for the chemical-induced HCC development in the mouse model

**Fig. 2:**
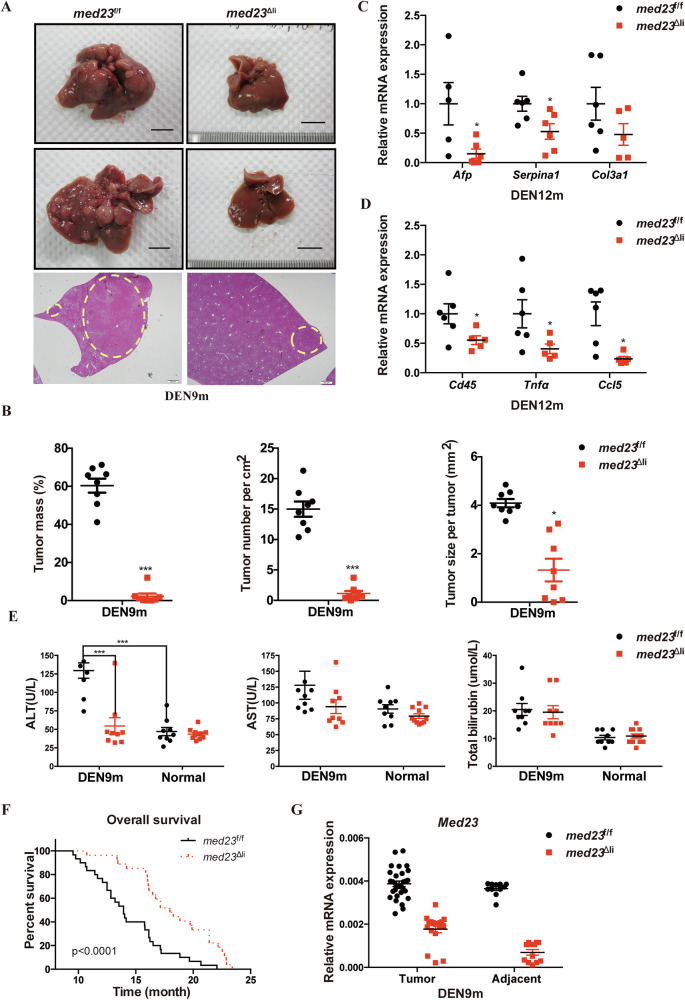
Analysis of liver cancer development in *med23*^f/f^ and *med23*^Δli^ mice after long-term administration of DEN. **A** Representative livers from DEN-injected *med23*^f/f^ and *med23*^Δli^ mice 9 months after DEN injection were shown. Liver sections from DEN-injected mice were stained with H&E, and representative pictures were shown. **B** Tumor mass, tumor number per cm^2^, and tumor size per tumor of DEN-induced HCCs in *med23*^f/f^ and *med23*^Δli^ mice were quantified (*n* = 8 per group). **C**, **D** Relative expression levels of *Afp*, *Serpina1*, *Col3a1*, *Cd45*, *Tnfα*, and *Ccl5* in tumors of *med23*^f/f^ and *med23*^Δli^ mice 12 months after DEN injection were analyzed by RT-PCR. The expression was normalized to *β-Actin* (*n* = 5–6 per group). **E** The amounts of alanine transaminase (ALT), aspartate transaminase (AST), and total bilirubin (T-BIL) in serum of untreated (normal) or DEN-treated mice at 9 months of age (DEN9m, *n* = 9 per group; Normal, *med23*^f/f^, *n* = 9, *med23*^Δli^, *n* = 11–12). **F** The overall survival curve of tumor-burdened *med23*^f/f^ and *med23*^Δli^ mice (*med23*^f/f^, *n* = 30, *med23*^Δli^, *n* = 27). **G** Relative expression levels of *Med23* in tumors and adjacent tissues of *med23*^f/f^ and *med23*^Δli^ mice 9 months after DEN injection were analyzed by RT-PCR. The expression was normalized to *β-Actin* (Adjacent, *med23*^f/f^, *n* = 11, *med23*^Δli^, *n* = 12; For tumors, *med23*^f/f^, *n* = 30, *med23*^Δli^, *n* = 18). Data are presented as mean ± SEM. Statistical significance was determined using unpaired Student’s *t* test. *P < 0.05, ***P < 0.001.

To explore the role of MED23 in HCC development in vivo, we utilized a genetically engineered mouse model by crossing *Med23*-floxed (*med23*^f/f^) mice with *Alb*-cre mice, in which *Med23* is constitutively ablated in hepatocytes [35, 36]. No histological abnormalities were observed in the liver of mice with hepatic deletion of *Med23* (hereafter *med23*^Δli^) under steady state, suggesting that *Med23* is dispensable for normal mouse liver development [38]. Single dose of carcinogen diethylnitrosamine (DEN) was subjected to male mice of postnatal day 14 to induce HCC development [10]. After 9-months induction, these mice were sacrificed and analyzed as previously reported [38]. Macroscopic examination and hematoxylin & eosin (H&E) staining revealed that control littermates (*med23*^f/f^) developed numerous hepatic tumors, whereas *med23*^Δli^ mice exhibited strong resistance to DEN-induced hepatic tumor formation, as evidenced by strikingly reduced tumor mass, tumor number, and tumor size (Fig. 2A, B). Consistently, the expression levels of HCC biomarkers *Afp* and *Serpina1*, together with fibrosis marker *Col3a1*, were reduced after *Med23* deletion at 12 months after DEN treatment (Fig. 2C).

Considering that HCC is an inflammation-associated malignant disease [41, 42], we analyzed the inflammation-related genes in isolated tumors. RT-PCR analysis demonstrated diminished liver inflammation in *med23*^Δli^ mice compared to control mice, as indicated by decreased expression levels of lymphocyte infiltration marker *Cd45* and pro-inflammatory factors *Tnfα* and *Ccl5* (Fig. 2D). Consistently, serum alanine transaminase (ALT) secretion, a marker of liver injury, was significantly decreased after *Med23* ablation, though the levels of aspartate transaminase (AST) and total bilirubin (T-BIL) were comparable (Fig. 2E), suggesting that *Med23* deficiency protects against long-term DEN-induced liver injury. Long-term DEN exposure caused loss of body weight accompanied with elevated liver/body weight ratio as a consequence of the burden of multiple tumors in control mice, which were completely prevented by *Med23* deletion (Supplementary Fig. 2A). In line with the reduced hepatic tumor incidence, DEN-treated *med23*^Δli^ mice displayed prolonged survival compared to *med23*^f/f^ mice (Fig. 2F).

Interestingly, when we analyzed the sporadic smaller tumors appeared in *med23*^Δli^ mouse livers, it turned out that these tumors showed about 50% *Med23* expression compared with those tumors in *med23*^f/f^ mice, suggesting that the sporadic tumors in KO livers might be due to the insufficient *Med23* deletion (Fig. 2G; Supplementary Fig. 2B). In light of this, *Med23* was hardly detectable in the neighboring non-tumor tissues of *med23*^Δli^ livers (Fig. 2G; Supplementary Fig. 2B), suggesting that *Med23*-null hepatocytes are resistant to malignant transformation by DEN. Taken together, these data demonstrate that hepatic *Med23* inactivation effectively abolishes chemically induced HCC development.

### Inducible ablation of *Med23* attenuates advanced HCC progression

**Fig. 3:**
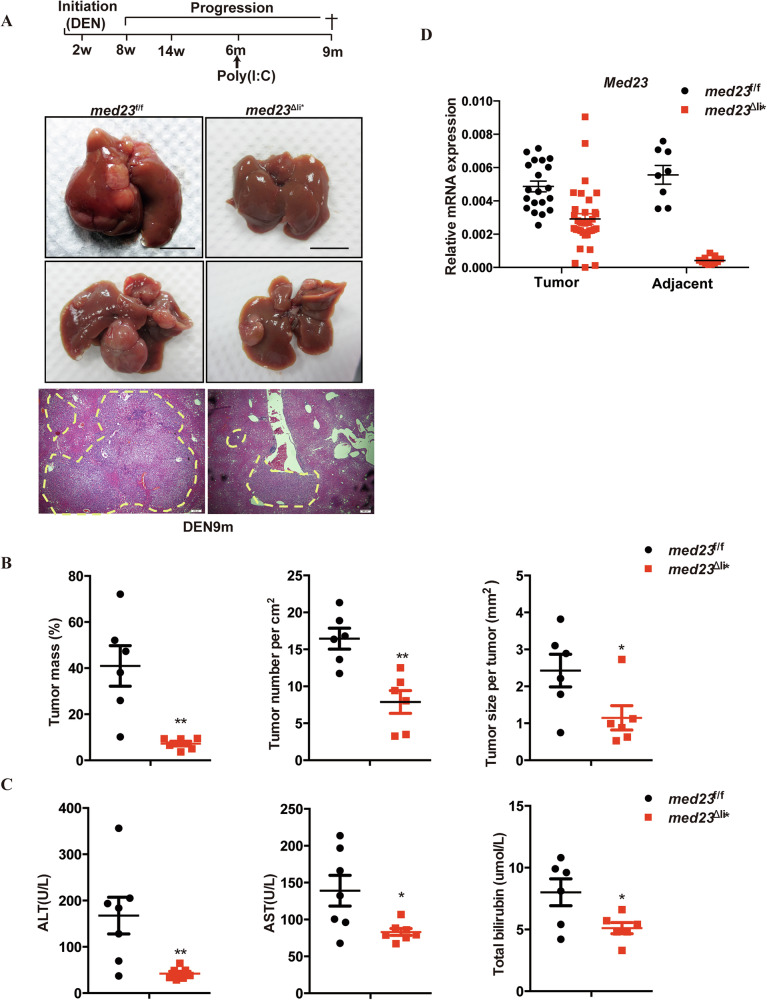
Analysis of liver cancer development in *med23*^f/f^ and *med23*^Δli^* mice after DEN administration. **A** Strategy to delete *Med23* in liver and tumor induction. Representative livers from DEN-injected *med23*^f/f^ and *med23*^Δli^* mice 9 months after DEN injection were shown. Liver sections from DEN-injected *med23*^f/f^ and *med23*^Δli^* mice were stained with H&E, and representative pictures were shown. **B** Tumor mass, tumor number per cm^2^, and tumor size per tumor of DEN-induced HCCs in *med23*^f/f^ and *med23*^Δli^* mice were quantified (*n* = 6 per group). **C** The amounts of ALT, AST, and T-BIL in serum of DEN-treated mice at 9 months of age (ALT and AST, *n* = 7 per group; T-BIL, *n* = 6 per group). **D** Relative expression levels of *Med23* in tumors and adjacent tissues of *med23*^f/f^ and *med23*^Δli^* mice 9 months after DEN injection were analyzed by RT-PCR. The expression was normalized to *β-Actin* (Adjacent, *med23*^f/f^, *n* = 8, *med23*^Δli^*, *n* = 11; For tumors, *med23*^f/f^, *n* = 20, *med23*^Δli^*, *n* = 33). Data are presented as mean ± SEM. Statistical significance was determined using unpaired Student’s *t* test. *P < 0.05, ***P < 0.001.

Hepatocarcinogenesis involves multi-stage progression including initiation, promotion, and progression. To investigate the role of *MED23* at different stages of liver cancer development, we crossed *med23*^f/f^ mice with inducible *Mx-Cre* transgenic mice [43] (termed *med23*^Δli^*), in which *Med23* can be efficiently deleted in liver and spleen but not in most other tissues upon poly(I:C) injection (Supplementary Fig. 3A). Compared to control mice, *med23*^Δli^* mice showed normal development without apparent abnormalities or defects in liver and body size after the short-term or long-term poly(I:C) injection (Supplementary Fig. 3B). Histological analysis also revealed undetectable alteration between livers of *med23*^f/f^ and *med23*^Δli^* mice (Supplementary Fig. 3C). Mice were subjected intraperitoneally with single dose of DEN on day 14, followed by poly(I:C) injection at 6 months when the carcinogenesis was supposed to be in progression stage [38], and the tumor development was assessed at 9 months (Fig. 3A). Similar to the constitutive Med23-deletion, poly(I:C)-induced *Med23* ablation dramatically suppressed hepatocarcinogenesis as shown by the representative images of *med23*^Δli^* and *med23*^f/f^ livers (Fig. 3A). Quantitative analysis revealed reduced tumor mass, tumor number, and tumor size in *med23*^Δli^* mice compared to *med23*^f/f^ mice (Fig. 3B). *Med23*^Δli^* mice also exhibited reduced liver weight and liver/body weight ratio due to the reduced tumor burden compared to *med23*^f/f^ mice (Supplementary Fig. 3D). As expected, lower serum ALT, AST, and T-BIL levels were observed in *Med23*^Δli*^ mice compared to control mice following DEN and poly(I:C) treatment (Fig. 3C), suggesting that hepatocyte-specific Med23 ablation mitigates DEN-induced liver injury. Consistent with findings in the *med23*^Δli^ model, although tumors from *med23*^Δli^* mice exhibited reduced *Med23* expression compared with tumors from *med23*^f/f^ mice, they still displayed higher mRNA levels of *Med23* than the surrounding normal liver tissues, where *Med23* mRNA was barely detectable (Fig. 3D). Together with the observations in the *med23*^Δli^ model, these findings demonstrate that Mediator *Med23* is required for DEN-induced hepatocarcinogenesis, highlighting that targeting *Med23* could effectively impede liver cancer development.

### *Med23* ablation leads to increased apoptosis but reduced compensatory proliferation in acute DEN-treated liver

**Fig. 4:**
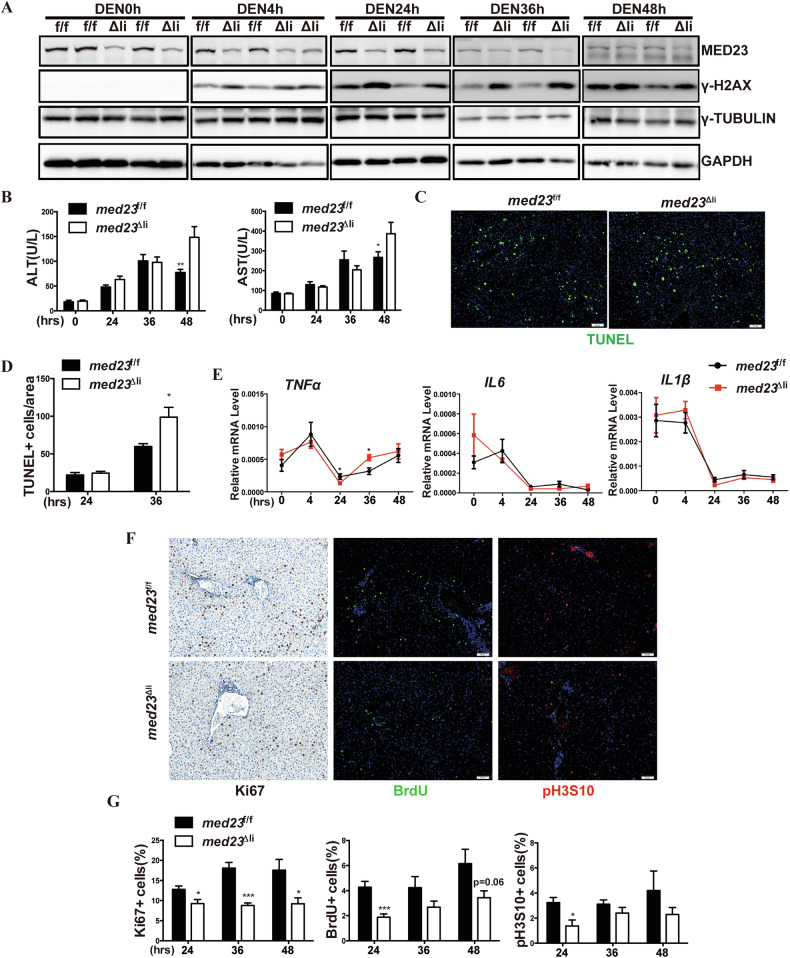
*Med23* ablation leads to increased apoptosis but compromised compensatory proliferation during initiation stage of DEN induction. **A** Immunoblot analysis of MED23, γ-H2AX, γ-TUBULIN, and GAPDH in liver tissues from *med23*^f/f^ and *med23*^Δli^ mice at indicated time after DEN treatment. **B** Analysis of ALT and AST in 2-week-old *med23*^f/f^ and *med23*^Δli^ mice after acute DEN treatment (*n* = 9–13 per group). **C, D** Representative views of TUNEL staining in the liver sections of *med23*^f/f^ and *med23*^Δli^ mice 36 h after DEN treatment (**C**), and quantification of TUNEL-positive cells from the liver sections of *med23*^f/f^ and *med23*^Δli^ mice (**D**). **E** Relative expression levels of *Tnfα, Il6, and Il1β* in liver tissues after DEN injection. The expression was normalized to *β-Actin* (*n* = 4–10 per group). **F**, **G** Representative liver sections (24 h after DEN injection) of *med23*^f/f^ and *med23*^Δli^ mice that were immunochemically stained with Ki67, BrdU, and pH3S10 (**F**), and percentages of positive cells were quantified (**G**) (*n* = 5–8 per group). Data are presented as mean ± SEM. Statistical significance was determined using unpaired Student’s *t* test. *P < 0.05, **P < 0.01, ***P < 0.001.

To investigate the cellular effect of hepatic *Med23* ablation, we collected the paired liver samples of 14 postnatal day male mice after acute DEN injection, and confirmed that *Med23* was efficiently deleted throughout the livers of *med23*^Δli^ mice compared to control livers (Fig. 4A; Supplementary Fig. 4A). DEN exposure could cause DNA adducts formation and DNA damage in liver [10]. *Med23*^Δli^ livers exhibited aggravated DNA damage compared to *med23*^f/f^ livers, as indicated by increased γ-H2AX staining (Fig. 4A). DEN-induced liver injury was also significantly increased in *med23*^Δli^ mice, as indicated by increased ALT and AST secretion, at 48 h after the acute injection (Fig. 4B). Consistently, TUNEL assay revealed more apoptotic cells in livers of *med23*^Δli^ mice than those of *med23*^f/f^ after acute DEN injection for 36 h when the cell death seems to reach the peak (Fig. 4C, D). These results indicate that Med23 deficiency sensitizes hepatocytes to DEN-induced DNA damage, leading to increased apoptosis and more severe liver damage, as reflected by elevated ALT and AST levels.

Unlike the acute model, in the chronic model, acute DNA damage was not persistent, thus no longer the primary determinant of liver pathology. Instead, sustained compensatory proliferation contributed to increased tumor burden in *med23*^f/f^ mice and secondary liver injury, thereby resulting in elevated ALT and AST levels (Fig. 2E). Pro-inflammatory cytokines, including TNFα, IL6, and IL1β, are mainly produced by Kupffer cells following hepatocyte death and paracrine stimulation, and play a critical role in DEN-induced liver cancer [13]. RT-PCR analysis showed comparable levels of these cytokines between livers of *med23*^f/f^ and *med23*^Δli^ mice after acute DEN treatment (Fig. 4E). Similarly, the expression of early response genes *C-jun* and *c-fos*, which are known to initiate liver cancer development [37], remained unchanged (Supplementary Fig. 4B). These results suggest that *MED23* ablation seems not to change the early stage of the DEN-induced response but may predominantly impact the later stage of HCC progression, implying that inhibiting MED23 might offer therapeutic benefits for advanced liver cancer.

Surviving hepatocytes undergo compensatory proliferation to maintain the liver’s homeostatic functions and may acquire carcinogen-induced mutagenesis, as a result of the enormous intrinsic regeneration capability [44, 45]. Ki67 staining revealed a remarkable reduction in proliferating hepatocytes in *med23*^Δli^ mice compared to *med23*^f/f^ mice (Fig. 4F, G), which was further confirmed by decreased 5’-bromo-2’-deoxyuridine (BrdU) incorporation and histone H3 phosphorylation staining (Fig. 4F, G). To further determine whether this proliferative defect is specific to DEN-induced injury or represents a general impairment of hepatocyte regeneration, we next applied the widely used two-thirds partial hepatectomy (PH) regeneration model to test whether *Med23* is also required for non-malignant proliferation of hepatocytes. *Med23*^Δli^ mice exhibited a modest reduction of Ki67-positive hepatocytes compared to *med23*^f/f^ mice at 96 h following operation (Supplementary Fig. 4C). Accordingly, *med23*^Δli^ mice failed to recover to the regular liver/body weight ratio of *med23*^f/f^ mice at 96 h after PH (Supplementary Fig. 4D). In addition, increased release of serum ALT and AST in *med23*^Δli^ mice indicated more severe liver injury than *med23*^f/f^ mice, which is in agreement with the DEN-induced HCC model (Supplementary Fig. 4E). Collectively, these data, together with the in vitro HCC cell lines results, support that MED23-deficiency suppresses DEN-induced HCC by inhibiting compensatory proliferation and enhancing apoptosis of hepatocytes.

### *Med23*^Δli^ livers exhibit increased ROS accumulation and reduced NQO1 protein upon DEN treatment

**Fig. 5:**
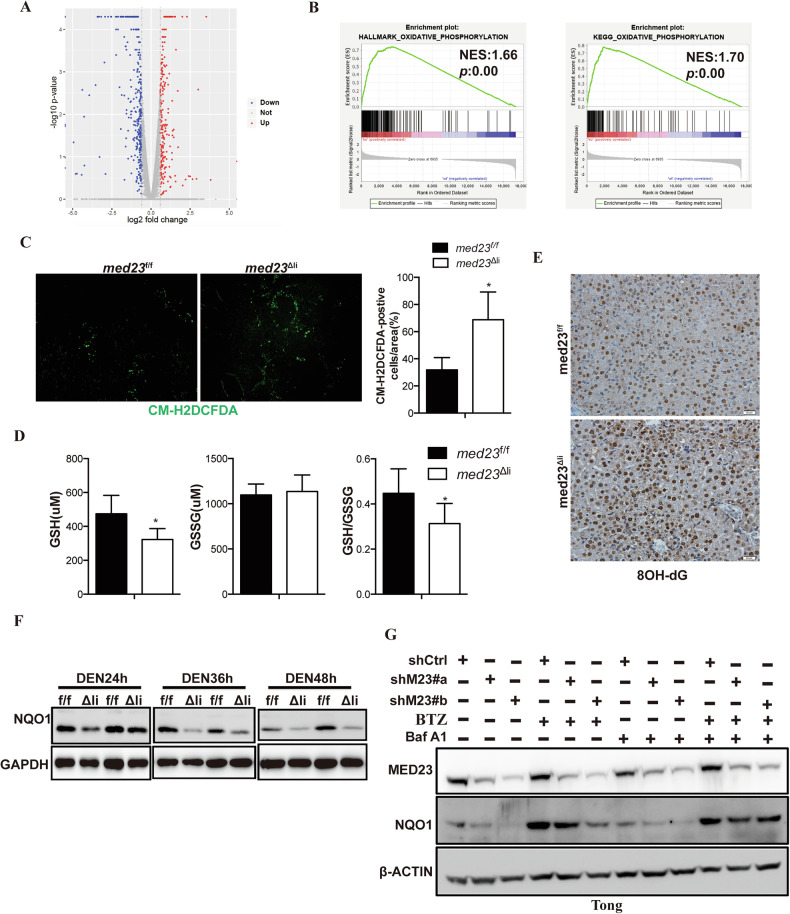
*Med23*^Δli^ livers exhibit increased ROS accumulation and reduced NQO1 protein. **A** Volcano plots depict gene expression changes between livers of *med23*^f/f^ and *med23*^Δli^. Significantly differential transcripts are highlighted in color and totaled in each direction (Fold change ≥ 1.5). Whole livers from *med23*^f/f^ and *med23*^Δli^ mice 48 h after DEN treatment were collected as samples for RNA-seq analysis. **B** GSEA analysis of RNA-Seq data from *med23*^f/f^ and *med23*^Δli^ mouse livers after acute administration of DEN for 48 h. **C** Liver sections (*n* = 5 per group) of *med23*^f/f^ and *med23*^Δli^ mice 48 h after acute DEN injection were stained with CM-H2DCFDA (left panels), and statistical analysis of CM-H2DCFDA-positive cells is presented (right panels) (*n* = 4 per group). **D** The content of reduced glutathione (GSH), oxidized glutathione (GSSG), and GSH/GSSG ratio in livers of *med23*^f/f^ and *med23*^Δli^ mice 24 h after DEN injection were measured and quatified. Representative results were shown (*n* = 6 per group). **E** 8OH-dG staining of liver sections from *med23*^f/f^ and *med23*^Δli^ mice after acute DEN injection. **F** Immunoblot analysis of NQO1 and GAPDH in liver tissues from *med23*^f/f^ and *med23*^Δli^ mice at indicated time after DEN treatment. **G** Immunoblot analysis of MED23, NQO1, and β-ACTIN in Tong cells transduced with retroviral shCtrl or shMED23 in the presence and absence of BTZ/Baf A1. Statistical significance was determined using unpaired Student’s *t* test. *P < 0.05, **P < 0.01.

To better understand the molecular mechanisms through which *Med23* deficiency affects apoptosis and proliferation, we carried out a genome-wide transcriptome analysis of whole livers from control and *med23*^Δli^ mice 48 h after DEN treatment. RNA-seq data analysis identified 351 genes that were down-regulated by more than 1.5-fold and 237 genes that were up-regulated by more than 1.5-fold in *med23*^Δli^ livers relative to controls (Fig. 5A). Interestingly, gene set enrichment analysis (GSEA) revealed that oxidative phosphorylation gene signatures were positively enriched in *Med23*-deficient livers (Fig. 5B). Consistent with these transcriptomic changes, staining of freshly frozen tissue sections showed increased ROS accumulation in acute DEN-treated *med23*^Δli^ mice compared with *med23*^f/f^ mice (Fig. 5C), which was further confirmed by flow cytometry analysis of isolated liver cells (Supplementary Fig. 5A). Consistently, liver reduced glutathione (GSH) levels, a major cellular antioxidant, were significantly decreased after *Med23* deletion (Fig. 5D). ROS accumulation could induce oxidative DNA damage, which can be specifically detected with antibody against 8-hydroxydeoxyguanosine (8-OHdG). Compared to *med23*^f/f^ livers, *Med23*^Δli^ livers displayed increased levels of 8-OHdG staining after acute DEN injection (Fig. 5E), indicating much more oxidative DNA damage was induced by *Med23* deficiency. In accordance with the results of mouse model, shRNA-mediated *MED23* knockdown similarly enhanced ROS accumulation in human HCC cells (Supplementary Fig. 5B), suggesting the cell autonomous regulation of ROS by MED23.

The environmental insults-stimulated aberrant ROS production can be detoxified by activating the NF-E2-related factor 2 (NRF2) signaling pathway and subsequently prompting the expression of anti-oxidative genes such as NQO1 and HO-1 [46, 47]. We found that the protein level of NQO1 was largely reduced in the livers of *med23*^Δli^ mice compared to controls after acute DEN treatment (Fig. 5F). Likewise, *MED23* knockdown decreased NQO1 protein levels in Tong (Fig. 5G; Supplementary Fig. 5C) and HepG2 cells (Supplementary Fig. 5D). However, *NQO1* mRNA levels were not reduced by *MED23*-deficiency either in mice (Supplementary Fig. 5E) or in human HCC cells (Supplementary Fig. 5F). Of note, the mRNA levels of *HO-1* and *NRF2* in livers with or without *Med23* were also unchanged (Supplementary Fig. 5E), which excludes the possibility of transcriptional control of NQO1 by MED23. Given these findings, we explored whether MED23 affects the protein stability of NQO1. Treatment with proteasome inhibitor (BTZ) either alone or in combination with lysosome inhibitor (Baf A1) restored NQO1 protein levels in *MED23* deficient cells (Fig. 5G), suggesting a possible protein degradation mechanism involved in NQO1 regulation by MED23. We also found that *NQO1* expression was upregulated in human HCC tumors compared to normal tissues (Supplementary Fig. 5G), and high NQO1 mRNA level was significantly associated with the poor prognosis of patients (Supplementary Fig. 5H). In sum, these findings show that MED23 prevents excessive ROS accumulation in DEN-treated livers, possibly through post-transcriptional regulation of the oncogenic protein NQO1.

### IGF2/IGF1R signaling pathway was attenuated by *Med23* ablation

**Fig. 6:**
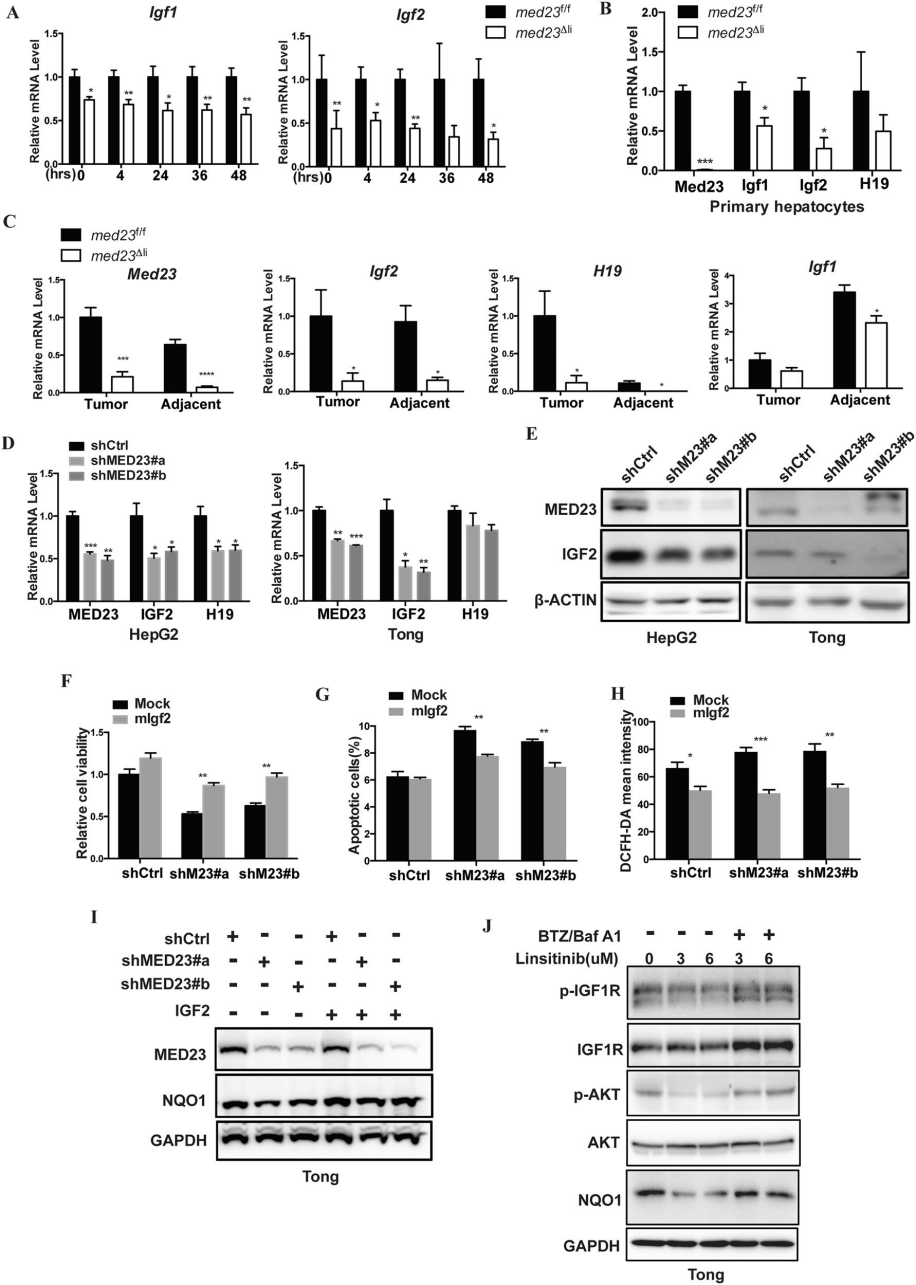
IGF2/IGF1R signaling pathway was compromised after *Med23* ablation. **A** Relative expression levels of *Igf1* and *Igf2* in liver tissues from *med23*^f/f^ and *med23*^Δli^ mice at indicated time after DEN treatment (*n* = 4–10 per group). **B** Relative expression levels of *Med23*, *Igf1*, *Igf2*, and *H19* in primary hepatocytes isolated from *med23*^f/f^ and *med23*^Δli^ mice (*n* = 3 per group). **C** Relative expression levels of *Med23*, *Igf2, H19*, and *Igf1* in liver tumor or adjacent tissue from *med23*^f/f^ and *med23*^Δli^ mice 12 months after DEN treatment (*n* = 5–8 per group). **D** Relative expression levels of *MED23, IGF2*, and *H19* in HepG2 and Tong cells transduced with retroviral shCtrl or shMED23 (*n* = 3–4 per group). **E** Immunoblot analysis of MED23, IGF2, and β-ACTIN in HepG2 and Tong cells transduced with retroviral shCtrl or shMED23. **F** Equal numbers of shCtrl or shMED23 Tong cells transduced with retroviral *mIgf2* were seeded into 6-well plate. The cells were washed with PBS and stained with crystal violet after cultured for 5 days. Statistical analysis of cell viability was presented (*n* = 3 per group). **G, H** ShCtrl and shMED23 Tong cells transduced with retroviral *mIgf2* were subjected to apoptosis analysis by PI/AnnexinV staining (**G**) or stained with CM-H2DCFDA (**H**), and statistical analysis was presented (*n* = 3 per group). **I** Immunoblot analysis of MED23, NQO1 and GAPDH in shCtrl or shMED23 Tong cells treated with recombinant IGF2 protein (100 ng/ml) for 4 days. The culture medium was renewed everyday. **J** Immunoblot analysis of p-IGF1R, IGF1R, p-AKT(S473), AKT, NQO1, and GAPDH in Tong cells treated with IGF1R inhibitor Linsitinib or BTZ/Baf A1. Data are presented as mean ± SEM. Statistical significance was determined using unpaired Student’s *t* test. *P < 0.05, **P < 0.01, ***P < 0.001.

To gain insight into how Med23 regulates the hepatic proliferative phenotype, we further analyzed the RNA-seq data and noticed an obvious reduction of the growth factors *Igf1* and *Igf2* in livers of *med23*^Δli^ mice as compared to *med23*^f/f^ mice (Supplementary Fig. 6A), which was confirmed by RT-PCR after acute DEN treatment (Fig. 6A). In contrast, the expression of other growth factors such as *Fgf21*, *Vegfa*, and *Hgf* remained unchanged upon *Med23* deletion (Supplementary Fig. 6A). Considering that the hepatocytes are the main source of these growth factors (*Igf1* and *Igf2*) [48], we isolated the primary hepatocytes from livers of untreated *med23*^f/f^ and *med23*^Δli^ mice and analyzed the expression levels of *Igf1* and *Igf2*. Both *Igf1* and *Igf2* were consistently downregulated in hepatocytes from *med23*^Δli^ mice (Fig. 6B). Interestingly, we noticed that H19 tended to be also declined in both the livers (Supplementary Fig. 6B) and hepatocytes derived from *med23*^Δli^ mice (Fig. 6B), suggesting that the expression of both H19 and Igf1/2 might be controlled similarly by Med23.

We then investigated whether the reduction of *Igf1*, *Igf2* and *H19* also occurs in *Med23*-deleted tumors. RT-PCR analysis showed that *Igf2* and *H19* were both dramatically reduced in tumors and adjacent normal tissues of *med23*^Δli^ mice compared to controls (Fig. 6C). Similar results were observed after acute deletion of *Med23* in *med23*^Δli^* mice burdened with tumors (Supplementary Fig. 6C). By contrast, *Igf1* was only reduced in normal liver tissues but not in tumors from *med23*^Δli^ mice or *med23*^Δli^* mice (Fig. 6C; Supplementary Fig. 6C), this underscores the specificity of Med23-IGF2 axis regulation in tumor cells.

Similar to that observed in mouse tumors, *MED23* knockdown also decreased *IGF2* mRNA and IGF2 protein levels in HCC cell lines such as HepG2 and Tong cells (Fig. 6D, E). To determine if IGF2 is indeed important in MED23-dependent cancer cell growth, we overexpressed *IGF2* in *MED23*-silenced Tong cells and observed that the ectopic *IGF2* partially rescued the phenotypes associated with Med23 deficiency, including impaired cell viability (Fig. 6F; Supplementary Fig. 6D), increased cell apoptosis (Fig. 6G), and accumulated ROS (Fig. 6H). When we examined the available database, there was a positive correlation for the mRNA levels of *MED23* and *IGF2 & H19* within tumors (Supplementary Fig. 6E). Given the established roles of IGF2 and H19 in promoting cell proliferation and tumorigenesis [19], these findings support the notion that MED23 promotes the development of HCC by regulating the expression of IGF2 and H19 in tumor cells. Notably, recombinant IGF2 supplementation also partially restored the decrease in NQO1 protein levels caused by *MED23* knockdown (Fig. 6I), which prompted us to investigate whether IGF2 may somehow regulate NQO1 expression. In order to further prove whether IGF2 affects the stability of NQO1, we treated the Tong and Huh7 cells with linsitinib, the inhibitor of IGF2 signaling (p-IGF1R). The results showed that linsitinib treatment largely decreased the protein level of NQO1 in Tong (Fig. 6J) and Huh7 (Supplementary Fig. 6F) cells, and this effect could be restored by BTZ/Baf A1 treatment together, which means the IGF2 signaling pathway promotes the stability of NQO1 by inhibiting the ubiquitin/proteasome pathway, thereby exerting an antioxidant function. After *MED23* knockdown, the decrease of IGF2 leads to decreased NQO1, impairing ROS clearance and resulting in the accumulation of ROS and cell death. In summary, these data suggest that MED23-IGF2 signaling may act upstream of NQO1-mediated ROS regulation, thus highlighting a MED23-IGF2-NQO1-ROS axis in the control of HCC development.

### MED23, cooperating with RFX5, controls *IGF2* expression through modulating enhancer function

**Fig. 7:**
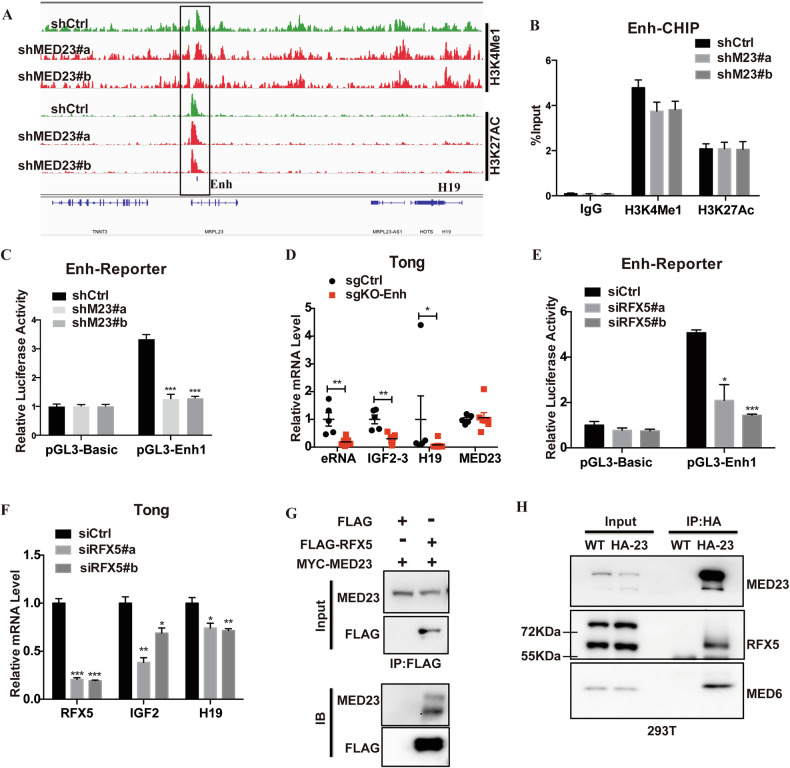
MED23 controls *IGF2* expression by modulating its enhancer function. **A** ChIP-seq analysis of H3K4me1 and H3K27ac enrichment around the *IGF2/H19* gene cluster in control and *MED23* knockdown Tong cells. **B** Verification of the ChIP-qPCR analysis at the Enh peak indicated in (**A**). ChIP assays were performed with an antibody specific for H3K4me1 and H3K27ac in control and *Med23*-deficient Tong cells, and then qPCR was performed (*n* = 3–4 per group). **C** Enhancer reporter assay in control and *Med23*-deficient Tong cells. The activity of Enh peak was measured by the ratio of firefly luciferase activity over GFP intensity (Firefly/GFP) and normalized to the empty vector control (*n* = 3 per group). **D** Relative *eRNA*, *IGF2*, *H19*, and *MED23* mRNA levels upon enhancer deletion are shown (*n* = 5–7 per group). **E** Enhancer reporter assay. Tong cells were transfected with siRNA against *RFX5*, and the activity of Enh peak was measured by the ratio of firefly luciferase activity over GFP intensity (Firefly/GFP) (*n* = 3 per group). **F** Relative expression levels of *RFX5*, *IGF2*, and *H19* in RFX5 knockdown Tong cells (*n* = 3 per group). **G** Co-immunoprecipitation (Co-IP) of MYC-MED23 with Flag-RFX5. Flag-RFX5 expressing plasmid was co-transfected with MYC-MED23 into 293T cells. Whole cell lysate was used for immunoprecipitation and then immunoblotting with indicated antibodies. **H** Physical interaction between endogenous RFX5 and MED23. Co-IP experiment was performed in 293T cells with a HA-tag that was knocked in MED23 locus. Whole cell lysate was used for immunoprecipitation with anti-HA beads, followed by detection with indicated antibodies by western blot. Data are presented as mean ± SEM. Statistical significance was determined using unpaired Student’s *t* test. *P < 0.05, **P < 0.01, ***P < 0.001.

To investigate how MED23 regulates *IGF2* transcription, we next performed a chromatin immunoprecipitation-sequencing (ChIP-seq) analysis to find out if epigenetics and cis-elements contribute to MED23 mediated regulation of IGF2. Guided by the ChIP-seq profiles of H3K4me1 and H3K27ac around the *IGF2/H19* locus in Tong cells, we identified a putative enhancer downstream of the *H19* transcript (Fig. 7A). In line with previous observations, analysis of a published ChIP-seq dataset revealed strong H3K27ac enrichment at the same region in HepG2 cells (Supplementary Fig. 7A). We observed that the histone modification signals of H3K4me1, but not H3K27ac, were slightly reduced at this enhancer region (Enh) in *MED23*-depleted cells (Supplementary Fig. 7B). ChIP-qPCR was performed to confirm that H3K4me1 occupancy at the Enh region of *IGF2* tended to be reduced by *MED23* deficiency (Fig. 7B). We then cloned this Enh sequence (~2Kb) into a luciferase reporter (pGL3-Basic) and transfected it into Tong cells. Compared to the basal reporter, the enhancer-driven reporter showed much higher transcription activity, which was reduced at least two-fold by *MED23*-knockdown (Fig. 7C). To determine if this newly identified enhancer element does connect to the *IGF2/H19* expression, we managed to delete this enhancer region using CRISPR/Cas9 technology in Tong cells. Sequencing confirmed a ~1.7 kb deletion flanking the identified enhancer peak (Supplementary Fig. 7C). Importantly, mRNA and eRNA levels of *IGF2* and *H19* were both decreased after deleting the Enh region, while *MED23* mRNA was not changed (Fig. 7D). Together, these observations suggest that MED23 likely controls the activity of the newly defined *IGF2/H19* enhancer.

The cooperative binding of transcription factors (TFs), Mediator complex, and other epigenetic regulators promotes long-range chromatin interactions between enhancers and promoters. We next sought to identify potential TFs that bind to this enhancer region. Bioinformatics analysis revealed four TFs (BRCA1, MZF1, RFX5, and ZNF354C) potentially binding at this enhancer region (Supplementary Fig. 7D). RFX5 ChIP-seq data of HepG2 cells from the ENCODE project revealed that RFX5 specifically binds to this IGF2 enhancer region (Supplementary Fig. 7A), and in addition, the RFX5-binding peaks happened to be largely overlapped with H3K27ac peaks (16431/34444) genome-wide, implying its participation in enhancer function in general (Supplementary Fig. 7E). Importantly, *RFX5* silencing attenuated the Enh reporter activity in Tong cells (Fig. 7E), and reduced the expression of *IGF2* and *H19* in both Tong cells (Fig. 7F) and HepG2 cells (Supplementary Fig. 7F); whereas transient *RFX5* overexpression upregulated *IGF2* and *H19* mRNAs in Tong cells (Supplementary Fig. 7G). These results demonstrate that RFX5 binds to this newly identified enhancer to control the expression of *IGF2* and *H19*.

As MED23 may regulate the activity of RFX5, we then tested the possibility if there is a direct physical association between RFX5 and MED23. A Co-immunoprecipitation (Co-IP) assay in 293T cells co-transfected with tagged *RFX5* and *MED23* confirmed the interaction between MED23 and RFX5 (Fig. 7G). Consistently, endogenous MED23 (with a knock-in HA-tag) and RFX5 interacted in 293T cells (Fig. 7H). Antibody to another Mediator kinase subunit CDK8 can readily pull down RFX5 as well as Mediator components (Supplementary Fig. 7H). This evidence strongly supports that Mediator complex may control the activity of RFX5 through protein-protein interaction.

Collectively, our data suggest a model that the RFX5, a putative oncogenic transcription factor in HCC, binds to the newly-identified Enh enhancer region and cooperates with Mediator MED23 to control *IGF2* and *H19* expression, thereby promoting hepatocarcinogenesis (Supplementary Fig. 8).

## Discussion

Carcinogenesis involves genetic alterations that drive oncogene activation and tumor suppressor inactivation, ultimately disrupting transcriptional regulation. Over the past decades, multiple transcriptional regulators have been identified in HCC. For example, c-JUN has been shown to be selectively required during the early-stage of HCC development [37, 49] whereas ATF4 mitigates hepatocyte death under stress by inhibiting ferroptosis, thereby delaying HCC progression [50]. However, targeted or pharmacological inhibition of transcription factors has proven challenging, possibly due to their indispensable roles in normal organ function. In contrast, transcriptional cofactors present promising therapeutic opportunities. Notably, the steroid receptor coactivator (SRC) stimulator MCB-613 selectively induces cancer cell death by disrupting cellular homeostasis [51]. Considering the pivotal role of Mediator MED23 in hepatic metabolic disorders and liver fibrosis, which are strongly associated with hepatocarcinogenesis, this study identified MED23 as a novel regulator of liver cancer development in murine models and human cancer cells. Mechanistic investigations revealed that MED23 supports the chemical-induced HCC progression by suppressing ROS-mediated cell death and promoting compensatory proliferation. Integrative analysis has therefore established the key importance of the MED23-IGF2-NQO1 axis in liver cancer development, suggesting potential targets for therapeutic intervention. Although MED23 may regulate other downstream signaling pathways, our findings provide functional evidence linking it to IGF2 and NQO1 regulation in liver cancer. Further studies should further clarify the specific contributions of these pathways to hepatocarcinogenesis.

The Mediator complex serves as an indispensable transcriptional coactivator [29]. Emerging evidence suggests that specific Mediator subunits exhibit cancer-specific dysregulation, potentially shaping tumor transcriptomes [40]. Perner’s group systematically mapped Mediator components across malignancies, identifying CDK19 and CDK8 as prostate cancer-specific regulators of metastatic behavior [52, 53]. In addition to CDK8, the specific effect of MED1 in liver cancer has also been reported. Reddy’s group revealed that there was a paradoxical selection for MED1+ tumor cells in MED1-deficient livers during chemical-induced hepatocarcinogenesis [54], a phenomenon mirroring our observations in *med23*^Δli^ models. These models collectively indicate that hepatocyte resistance to transformation depends on intact Mediator components, although the molecular mechanisms governing MED1 and MED23 functions appear to operate through distinct pathways. Of note, it is well-established that MED1 can regulate multiple biological processes through distinctive interactions with different nuclear receptor family members (e.g., AR, ER), whereas MED23 is primarily targeted by MAPK-activated transcription factors (e.g., Elk1, Ets1). In the future, dissecting the shared and distinctive mechanisms of MED1 vs MED23 in directing chemical-induced HCC could be further explored.

Using in vivo and in vitro systems, we previously demonstrated that MED23 deletion negatively impacts key monocyte chemotaxis gene signatures, leading to enhanced hepatic stellate cells (HSCs) activation and excessive extracellular matrix (ECM) deposition [36]. Additionally, while MED23 knockout inhibits lung cancer cell proliferation in vitro [55], it paradoxically downregulates MHC class I subunit B2M in vivo, facilitating immune evasion and tumor progression [56]. These findings underscore the sophisticated functions of MED23 and emphasize the necessity of investigating its tissue-specific phenotypic effects. In this study, ChIP-seq analysis revealed that MED23 colocalizes with active histone marks and transcription factors, suggesting its role in transcriptional activation. Notably, MED23 depletion led to diminished enhancer activity and reduced IGF2/H19 expression, reinforcing the concept that enhancer-promoter interactions rely on transcription factors (TFs), Mediator, and cohesin recruitment [57, 58]. Regarding the involvement of enhancer-promoter looping regulators, Paxillin, a focal adhesion protein, has been implicated in enhancer-promoter looping at IGF2/H19 regulatory elements, underscoring the structural complexity of transcriptional regulation [59]. A recent work in breast cancer also demonstrated that high-order assemblies of transcription factors, such as ERα and GATA3, can trigger genome-wide enhancer reprogramming, thereby promoting tumor phenotypic plasticity and therapy resistance [60]. In our case, Regulatory Factor X5 (RFX5), a winged-helix transcription factor essential for MHCII gene regulation [61, 62], was significantly enriched in the IGF2/H19 enhancer region, with ChIP-seq analysis revealing a substantial overlap (47.7%) between RFX5 and H3K27ac peaks. RFX5 knockdown reduced IGF2/H19 enhancer activity, suggesting its role in promoting enhancer function. Given the well-established oncogenic role of IGF2 in HCC [23], these findings indicate that MED23 and RFX5 act as key coregulators, facilitating IGF2/H19 expression through enhancer regulation. Additionally, as the Mediator complex has been identified as a transducer of activating non-coding RNAs (ncRNAs) [63], future research on whether MED23 orchestrates eRNA transcription to further elucidate its mechanistic role in enhancer regulation should be rather interesting. This observation aligns with recent studies showing that Mediator-regulated enhancer-promoter loops orchestrate lineage-specific transcriptional programs in cancer [64]. Overall, we propose a mechanistic model, in which Mediator MED23 acts as a key regulator and coordinates with RFX5, eRNAs, and Pol II transcriptional machinery to modulate enhancer activity for IGF2/H19 transcription (Supplementary Fig. 8).

Taken together, our findings highlight the critical role of MED23 in HCC and provide a mechanistic framework linking MED23 to hepatic enhancer regulation. Given its functional specificity, liver-directed MED23 inhibition emerges as a compelling strategy for combating both obesity and hepatocarcinogenesis.

## Supplementary information

Figure S1

Figure S2

Figure S3

Figure S4

Figure S5

Figure S6

Figure S7

Figure S8

aj-checklist.

Supplementary figure legend and Table

western

## Data Availability

All relevant data are available within the article and its supplementary information files or can be obtained from the corresponding author upon reasonable request. The RNA-seq and ChIP-seq data have been deposited in the GEO database under accession number GSE295431, GSE295432.
