# Supplementary material for: Targeting MED23 inhibits hepatocellular carcinoma development by suppressing compensatory proliferation and facilitating ROS-mediated cell death
Source: Cell Death Dis. 2025 Dec 24;17(1):131. doi: 10.1038/s41419-025-08348-8 (PMC12848160; doi:10.1038/s41419-025-08348-8)
Supplement: Supplementary file 10 — Supplementary figure legend and Table [file 41419_2025_8348_MOESM10_ESM.docx]

**Figure legend**

**Supplementary Fig. 1 MED23 is upregulated in human HCC tumors and regulates proliferation and apoptosis of HCC cells.** (A) Relative *MED23* expression levels in human HCC tumor and adjacent non-tumor samples from the public datasets including GSE17856 (Non-tumor, n = 40; Tumor, n = 40) and GSE36376 (Non-tumor, n = 193; Tumor, n = 240). (B) Tong cells transduced with retroviral shCtrl or shMED23 were plated and incubated with EdU, and then stained. Representative results were shown. (C) Tong cells transduced with retroviral shCtrl or shMED23 were subjected to PI/AnnexinV staining to detect apoptotic cells, and representative results were shown. Data are presented as mean ± SEM. The Shapiro-Wilk test was applied to test the data for normality. Statistical significance was determined using unpaired Student’s t-test. ****P* < 0.001.

**Supplementary Fig. 2 Analysis of liver cancer development in *med23*^f/f^ and *med23*^Δli^ mice after long-term administration of DEN.** (A) Analysis of liver weight, body weight, and liver/body weight in untreated (normal) or DEN-treated mice at 9 months of age (DEN9m, *med23*f/f, n = 11, *med23*^Δli^, n = 12; Normal, *med23*f/f, n = 9, *med23*^Δli^, n = 12). (B) Immunoblot analysis of MED23, γ-TUBULIN, β-ACTIN and GAPDH in tumor (T) and non-tumor (N) liver tissues from DEN-injected mice as well as liver tissues from untreated (normal) mice. Data are presented as mean ± SEM. Statistical significance was determined using unpaired Student’s t-test. **P* < 0.05, ****P* < 0.001.

**Supplementary Fig. 3 Analysis of liver cancer development in *med23*^f/f^ and *med23*^Δli^*** mice after DEN administration.** (A) Immunoblot analysis of MED23, γ-TUBULIN, β-ACTIN and GAPDH in liver, spleen, heart, kidney, lung, and muscle tissues from *med23*^f/f^ and *med23*^Δli^*** mice. (B) Analysis of body weight, liver/body weight, ALT, and AST in *med23*^f/f^ and *med23*^Δli^*** mice 2 weeks or 2 months after Poly(I:C) injection. (C) Liver sections from *med23*^f/f^ and *med23*^Δli^*** mice 2 weeks or 2 months after Poly(I:C) injection were stained with H&E, and representative pictures were shown. (D) Analysis of liver weight, body weight, and liver/body weight in untreated (normal) or DEN-treated *med23*^f/f^ and *med23*^Δli^*** mice at 9 months of age (DEN9m, *med23*f/f, n = 7, *med23*^Δli^***, n = 8; Normal, *med23*f/f, n = 4, *med23*^Δli^***, n = 5). Data are presented as mean ± SEM. Statistical significance was determined using unpaired Student’s t-test. ***P* < 0.01.

**Supplementary Fig. 4 *Med23* ablation leads to increased apoptosis but compromised compensatory proliferation during initiation stage of DEN induction.** (A-B) Relative expression levels of *Med23*, *c-Jun*, and *c-Fos* in liver tissues after DEN injection. The expression was normalized to *β-Actin* (n = 7-10 per group)*.* (C) Representative liver sections (96 hours after PH) of *med23*f/f and *med23*^Δli^ mice that were immunochemically stained with Ki67 and percentages of positive cells were quantified (n = 3 per group). (D) Analysis of liver/body weight in *med23*^f/f^ and *med23*^Δli^ mice 48 hours or 96 hours after PH (n = 3-5 per group). (E) Analysis of ALT and AST in *med23*^f/f^ and *med23*^Δli^ mice 96 hours after PH (n = 3 per group). Data are presented as mean ± SEM. Statistical significance was determined using unpaired Student’s t-test. **P* < 0.05, ****P* < 0.001.

**Supplementary Fig. 5 *Med23*^Δli^ livers exhibit increased ROS accumulation and reduced NQO1 protein.** (A) Primary liver cells of *med23*^f/f^ and *med23*^Δli^ mice 48 hours after acute DEN injection were stained with CM-H2DCFDA (left panels), and statistical analysis of CM-H2DCFDA-positive cells is presented (right panels) (n = 4 per group). (B) Tong cells transduced with retroviral shCtrl or shMED23 were stained with CM-H2DCFDA (left panels), and statistical analysis of CM-H2DCFDA-positive cells is presented (right panels). (C) Representative views of NQO1 staining of Tong cells transduced with retroviral shCtrl or shMED23. (D) Immunoblot analysis of MED23, NQO1, and β-ACTIN in HepG2 cells transduced with retroviral shCtrl or shMED23. (E) Relative expression levels of *Nqo1*, *HO-1*, and *NRF2* in liver tissues from *med23*^f/f^ and *med23*^Δli^ mice at indicated time after DEN treatment (n = 4-7 per group). (F) Relative expression levels of *MED23* and *NQO1* in Tong and HepG2 cells transduced with retroviral shCtrl or shMED23 (n = 3 per group). (G) Relative *NQO1* expression level in human HCC tumor and adjacent non-tumor samples. (H) Predicting the 5-year overall survival of HCC patients according to the expression level of *NQO1* (https://kmplot.com). Data are presented as mean ± SEM. The Shapiro-Wilk test was applied to test the data for normality. Statistical significance was determined using unpaired Student’s t-test. ***P* < 0.01, ****P* < 0.001.

**Supplementary Fig. 6 IGF2/IGF1R signaling pathway was compromised after *Med23* ablation.** (A) Volcano plots depict gene expression changes between livers of *med23*^f/f^ and *med23*^Δli^ 48 hours after DEN treatment. IGF2, IGF1, and other factors were added in the proper position. (B) Relative expression levels of *H19* in liver tissues from *med23*^f/f^ and *med23*^Δli^ mice at indicated time after DEN treatment (n = 6-9 per group). (C) *Med23*^f/f^ and *med23*^Δli^*** mice 9 months after DEN treatment were injected with one single PolyIC to delete *Med23*. Then 1 week later, the relative expression levels of *Med23*, *Igf2, H19*, *Igf1* in liver tumor or adjacent tissue were measured (n = 4-6 per group). (D) Equal numbers of shCtrl or shMED23 Tong cells transduced with retroviral *mIgf2* were seeded into 6-well plate. The cells were washed with PBS and stained with crystal violet after cultured for 5 days. Representative pictures were shown. (E) Co-expression analyses between MED23 and IGF2/H19 in 360 HCC patients/samples (https://www.cbioportal.org). (F) Immunoblot analysis of p-IGF1R, IGF1R, p-AKT(S473), AKT, NQO1, and GAPDH in Huh-7 cells treated with IGF1R inhibitor Linsitinib or BTZ/Baf A1. Data are presented as mean ± SEM. Statistical significance was determined using unpaired Student’s t-test. **P* < 0.05, ***P* < 0.01.

**Supplementary Fig. 7 MED23 controls *IGF2* expression by modulating its enhancer function.** (A) Analysis of H3K27ac, RFX5, FOXA1, and HNF4A ChIP-seq binding peak in HepG2 cells from ENCODE. (B) Relative quantification of the binding density of Enh peak shown in (Fig. 7A). (C) Representative picture of Sanger sequencing of PCR products of sgCtrl and sgKO-Enh samples in Tong cells. (D) Bioinformatics prediction of the potential binding transcription factors that binding to the Enh peak. (E) Overlap between RFX5-bound peaks and H3K27ac peaks genome-wide according to ChIP-seq data of HepG2 cells from the ENCODE project. (F) Relative expression levels of *RFX5*, *IGF2*, and *H19* in RFX5 knockdown HepG2 cells (n = 3 per group). (G) Relative expression levels of *RFX5*, *IGF2*, and *H19* in transient RFX5 overexpressed Tong cells (n = 3 per group). (H) Physical interaction between endogenous RFX5 and Mediator complex. Co-IP experiment was performed in Tong cells, and whole cell lysate was used for immunoprecipitation with anti-CDK8 antibody, followed by detection with indicated antibodies by western blot.

**Supplementary Fig. 8 A schematic model for Mediator MED23 in regulating IGF2/IGF1R signaling and NQO1 expression, and contributing to HCC development.**

**Supplementary Table S1. The sequences of siRNA oligonucleotides.**

| siRNA | sense (5’-3’) | antisense (5’-3’) |
| --- | --- | --- |
| siCtrl | UUCUCCGAACGUGUCACGUTT | ACGUGACACGUUCGGAGAATT |
| RFX5-Homo-#1 | GCAUAAGGAGGAAGACCUUTT | AAGGUCUUCCUCCUUAUGCTT |
| RFX5-Homo-#2 | CCUUAUCCCAGGAGCAUAATT | UUAUGCUCCUGGGAUAAGGTT |

**Supplementary Table S2. Primer sequences used in the RT-PCR analysis**

**(mouse).**

| Primer name | Sequence (5’-3’) |
| --- | --- |
| *Enh*-1-F | GTTACCGTGGAGGTGCTTGT |
| *Enh*-1-R | ACTGTCCTTGTTCCCACAGG |

F: forward primer, R: reverse primer.

**Supplementary Table S3. Primer sequences used in the RT-PCR analysis**

**(human).**

| Primer name | Sequence (5’-3’) |
| --- | --- |
| *Med23*-F | TCGGAAAATCATTGGAGGAG |
| *Med23*-R | CAATAGGCAGGCATTTCGTT |
| *c-Fos-*F | CCAGTCAAGAGCATCAGCAA |
| *c-Fos-*R | AAGTAGTGCAGCCCGGAGTA |
| *c-Jun-*F | TCCCCTATCGACATGGAGTC |
| *c-Jun-*R | TGAGTTGGCACCCACTGTTA |
| *Cd45-*F | CCAGCAGACAGGGTTGTTCT |
| *Cd45-*R | CGGGATAGATGCTGGCGATG |
| *Tnfa-*F | CGTCAGCCGATTTGCTATCT |
| *Tnfa-*R | CGGACTCCGCAAAGTCTAAG |
| *Il-6-*F | CTGCAAGAGACTTCCATCCAG |
| *Il-6-*R | AGTGGTATAGACAGGTCTGTTGG |
| *Il1β-*F | GCCCATCCTCTGTGACTCAT |
| *Il1β-*R | AGGCCACAGGTATTTTGTCG |
| *Ifnγ-*F | AACGCTACACACTGCATCT |
| *Ifnγ-*R | GAGCTCATTGAATGCTTGG |
| *Ccl5-*F | GCTGCTTTGCCTACCTCTCC |
| *Ccl5-*R | TCGAGTGACAAACACGACTGC |
| *Afp*-F | GCCATGAAGTGGATCACACC |
| *Afp*-R | CTCCTCGGTGGCTTCCGGAA |
| *Col3a1*-F | GGGGACCAGGGCGACCACT |
| *Col3a1*-R | CAGGTGAACCCGGCAAGAACG |
| *Serpine1*-F | ATCGAGGTAAACGAGAGCGG |
| *Serpine 1*-R | CCACTGTCAAGGCTCCATCA |
| *Igf1*-F | CTCTTCTACCTGGCGCTCTG |
| *Igf1*-R | GCAACACTCATCCACAATGC |
| *Igf2*-F | GTGCTGCATCGCTGCTTAC |
| *Igf2*-R | ACGTCCCTCTCGGACTTGG |
| *H19*-F | GTGTCACCAGAAGGGGAGTG |
| *H19*-R | AGTGCCTCATGGGAATGGTG |
| *NQO1*-F | AGGATGGGAGGTACTCGAATC |
| *NQO1*-R | AGGCGTCCTTCCTTATATGCTA |
| *NRF2*-F | TCTTGGAGTAAGTCGAGAAGTGT |
| *NRF2*-R | GTTGAAACTGAGCGAAAAAGGC |
| *HO-1*-F | AAGCCGAGAATGCTGAGTTCA |
| *HO-1*-R | GCCGTGTAGATATGGTACAAGGA |
| *Actin*-F | CTGGCTGGCCGGGACCTGACA |
| *Actin*-R | ACCGCTCGTTGCCAATAGTGATGA |

F: forward primer, R: reverse primer.

**Supplementary Table S4. Primer sequences used in the ChIP-qPCR analysis.**

| Primer name | Sequence (5’-3’) |
| --- | --- |
| *MED23*-F | GGGGGTCAGCAACTCAATAA |
| *MED23*-R | GCTCTGGGGAATTTTCCTTC |
| *IGF1*-F | TCTCTGAATCTTGGCTGCTG |
| *IGF1*-R | TTGGTGTGCTTCTTGACGAC |
| *IGF2*-F | GTGGCATCGTTGAGGAGTG |
| *IGF2*-R | CACGTCCCTCTCGGACTTG |
| *H19*-F | CGAGTGTGCGTGAGTGTGA |
| *H19*-R | GGCGTAATGGAATGCTTGA |
| *NQO1*-F | TTACTATGGGATGGGGTCCA |
| *NQO1*-R | TCTCCCATTTTTCAGGCAAC |
| *RFX5*-F | GATGAGCCTGATGCTAAGAGC |
| *RFX5*-R | GGGAGCTGAAGGTAGAGATACA |
| *ACTIN*-F | CTCCATCCTGGCCTCGCTGT |
| *ACTIN*-R | GCTGTCACCTTCACCGTTCC |

F: forward primer, R: reverse primer.

**Supplementary Table S5. The sequences of shRNA oligonucleotides.**

| shRNA | sequences |
| --- | --- |
| shCtrl | GTGCGCTGCTGGTGCCAAC |
| shMED23#a | GGAACAAACTGTATGGCTT |
| shMED23#b | GCAAGAGAGGTTATAGCAT |
