# Supplementary material for: Targeting MED23 inhibits hepatocellular carcinoma development by suppressing compensatory proliferation and facilitating ROS-mediated cell death
Source: Cell Death Dis. 2025 Dec 24;17(1):131. doi: 10.1038/s41419-025-08348-8 (PMC12848160; doi:10.1038/s41419-025-08348-8)

Fig1C-MED23

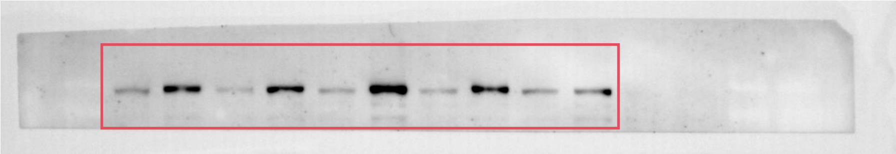

Fig1C-β-ACTIN-GAPDH

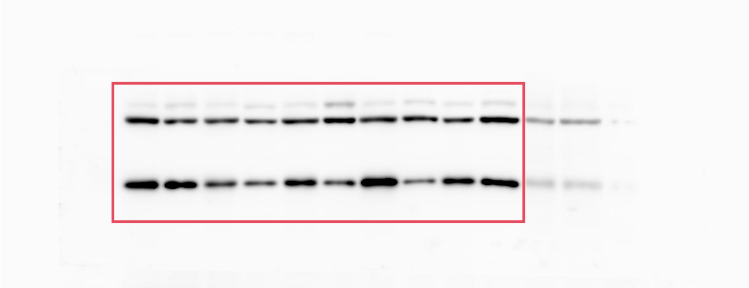

Fig1C-γ-TUBULIN

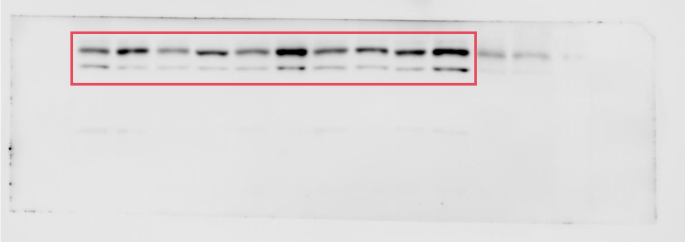

Fig1E-MED23

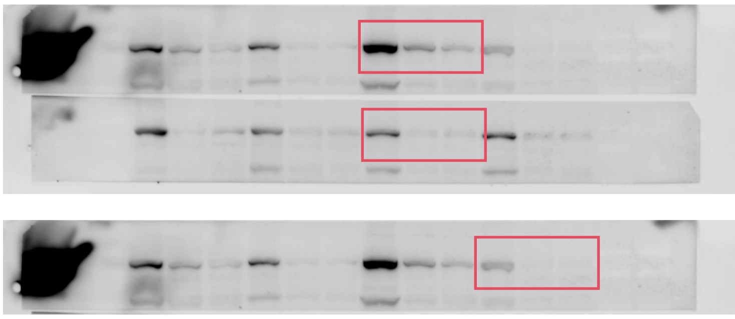

Fig1E-γ-TUBULIN

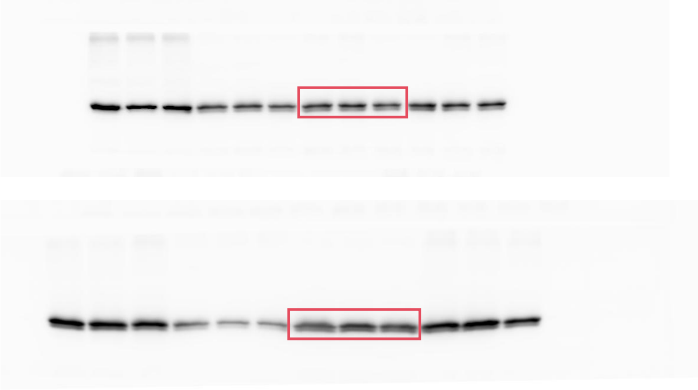

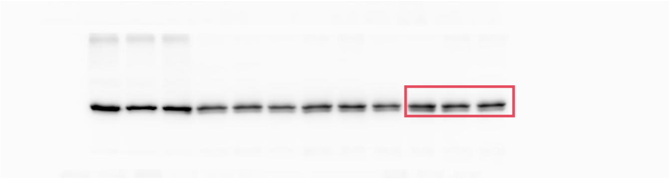

Fig4A-MED23

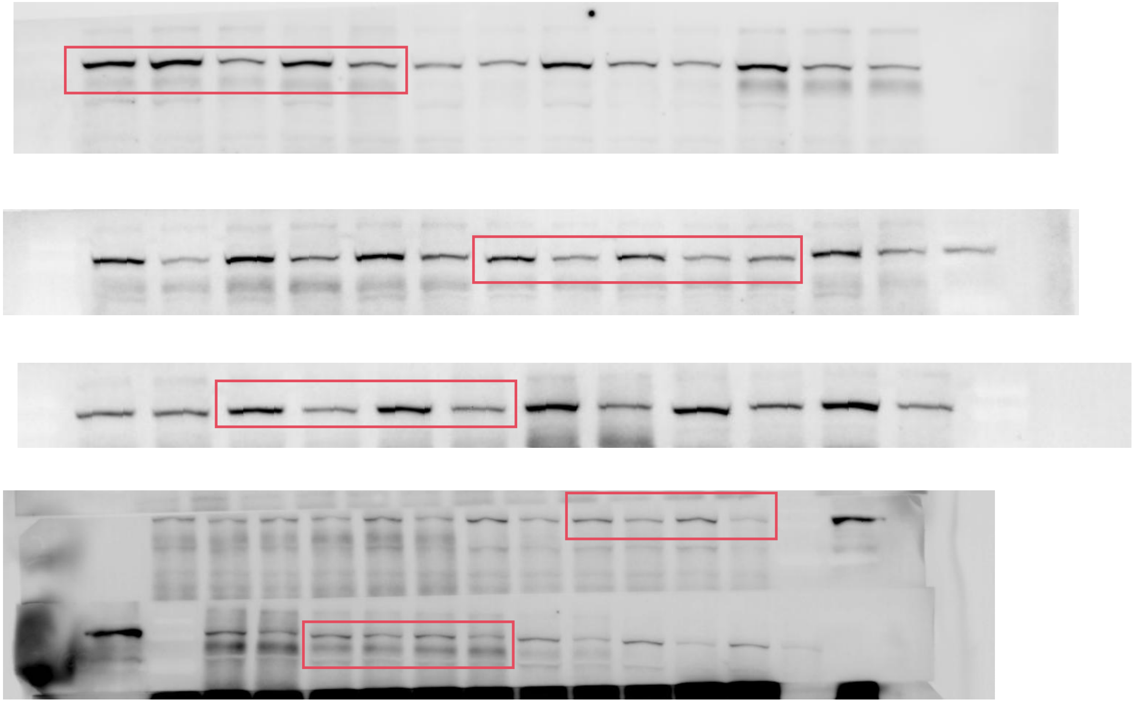

Fig4C-γ-H2AX

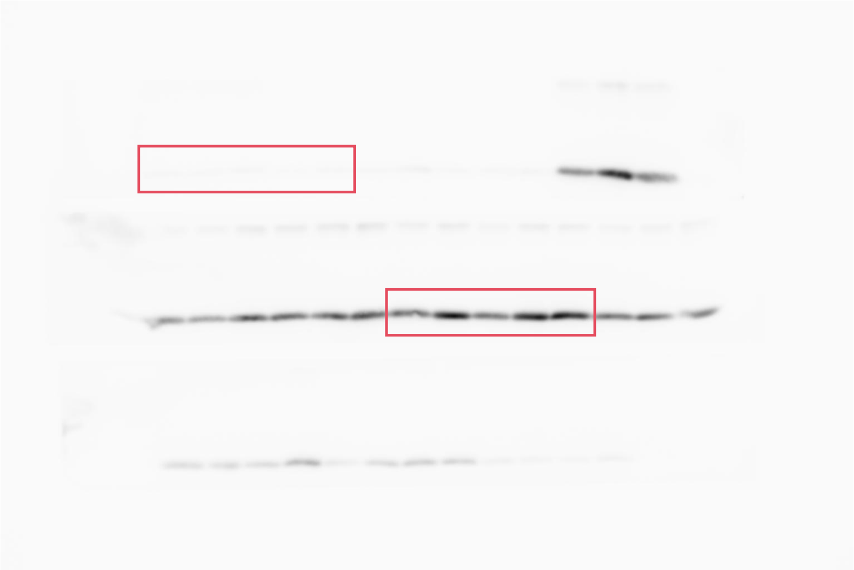

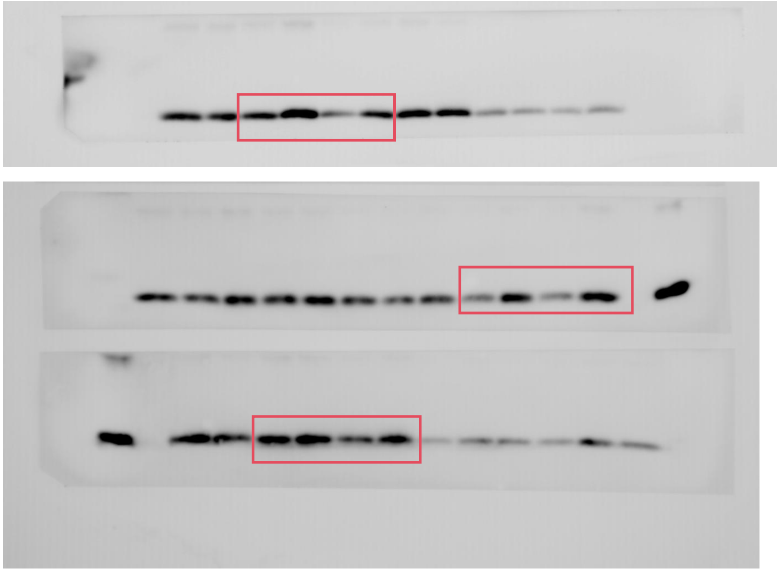

Fig4C-GAPDH

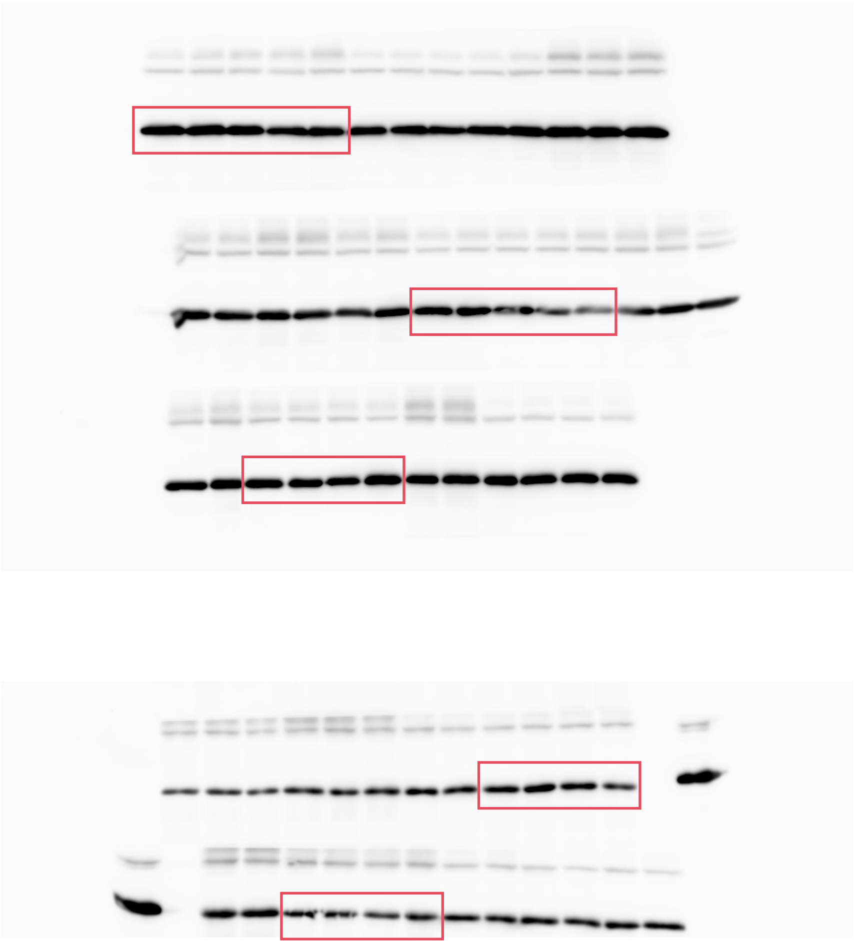

Fig4C-γ-TUBULIN

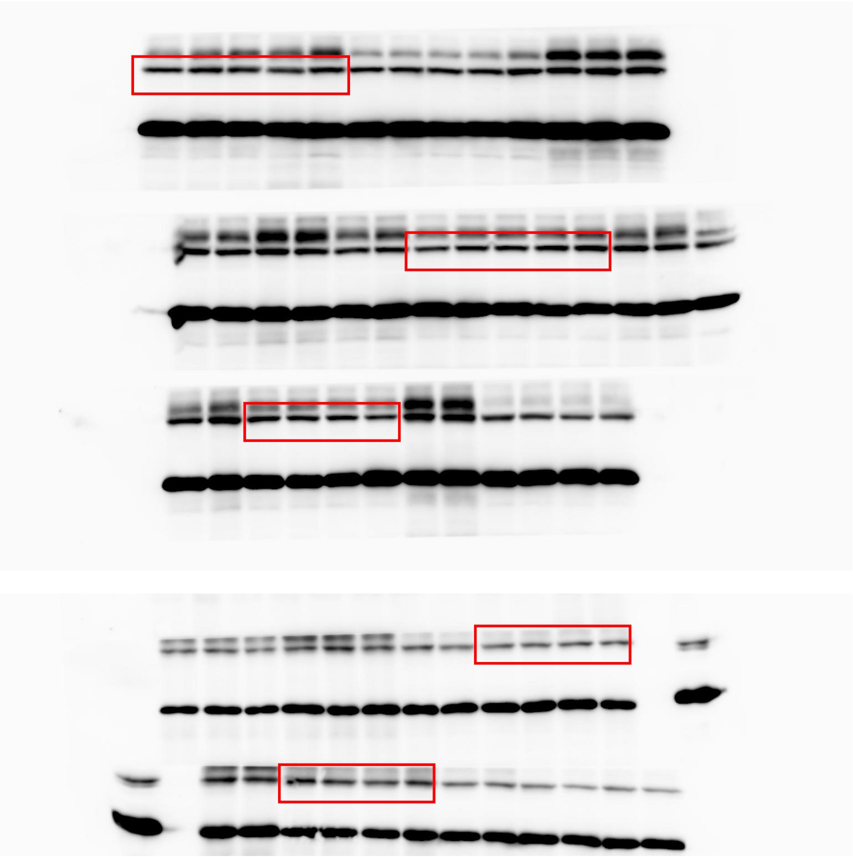

Fig5F-NQO1

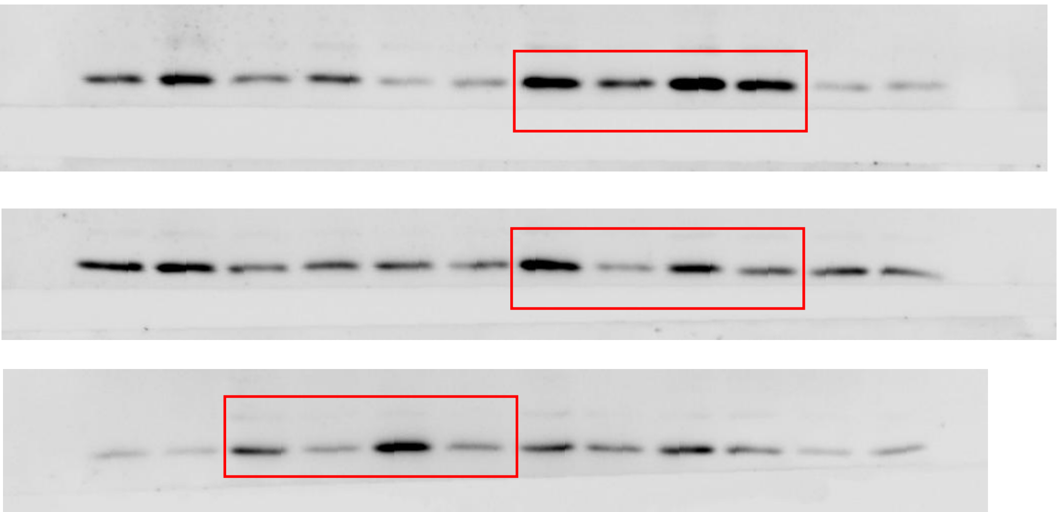

Fig5F-GAPDH

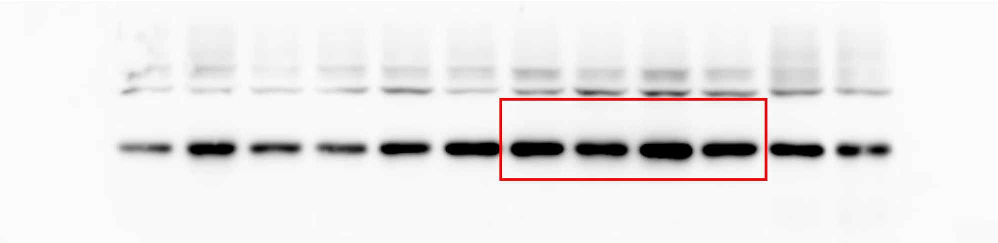

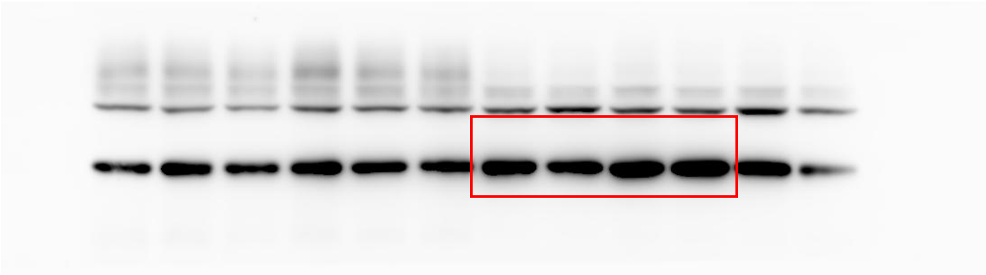

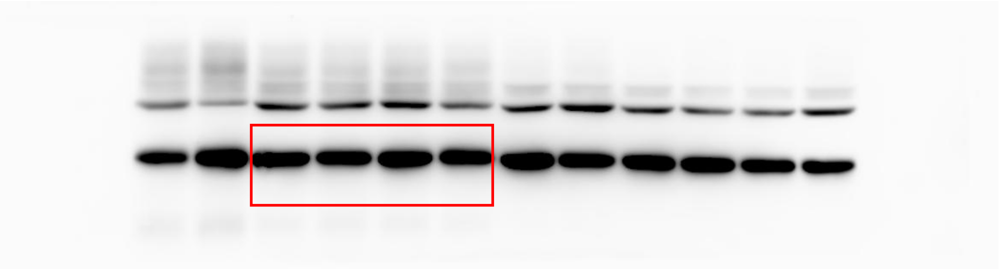

Fig5G-MED23

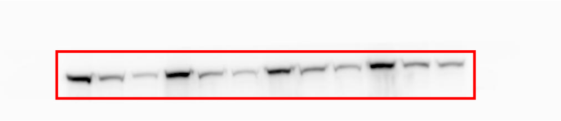

Fig5G-NQO1

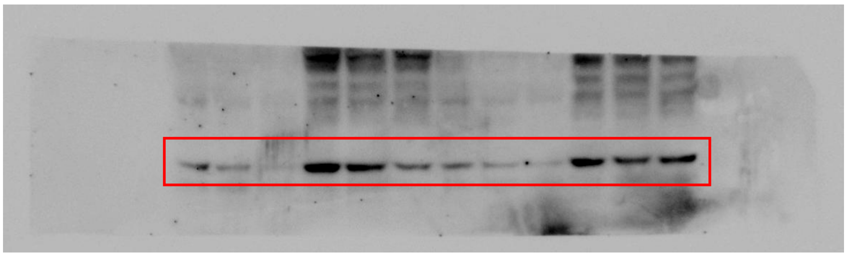

Fig5G-β-ACTIN

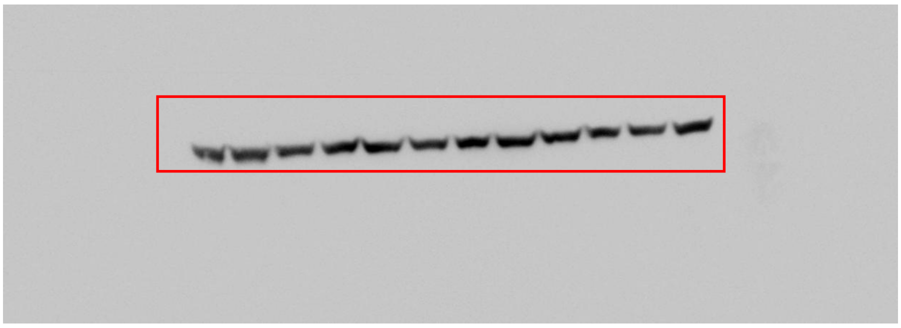

Fig6E-MED23

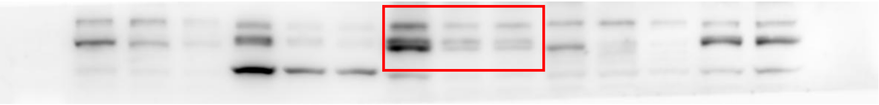

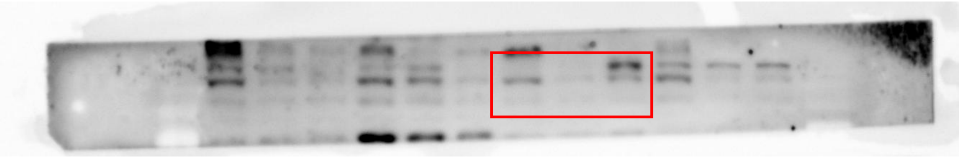

Fig6E-IGF2

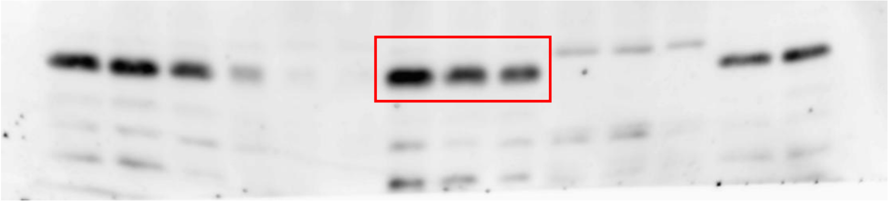

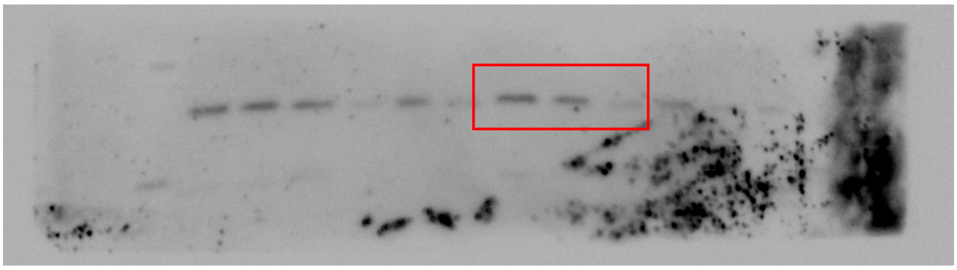

Fig6E-β-ACTIN

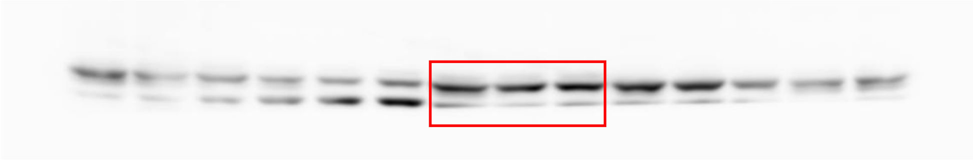

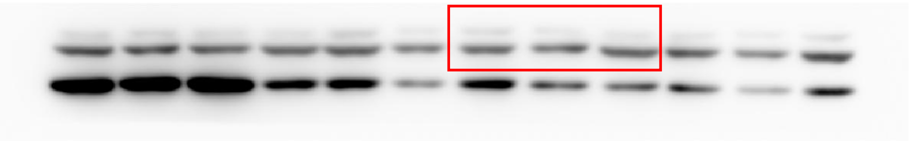

Fig6I-MED23

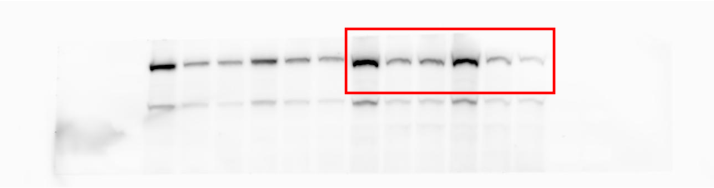

Fig6I-NQO1

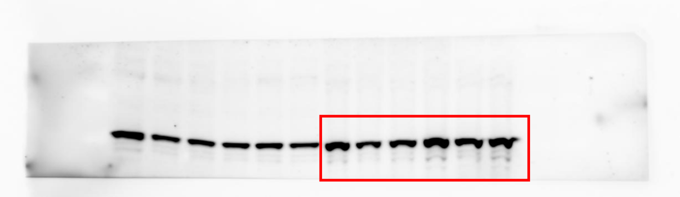

Fig6I-GAPDH

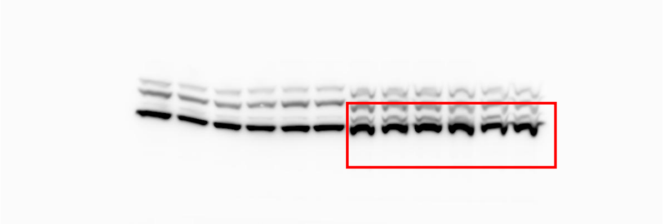

Fig6J-p-IGF1R

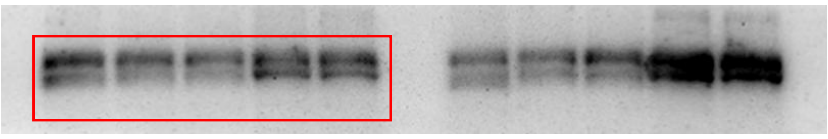

Fig6J-IGF1R

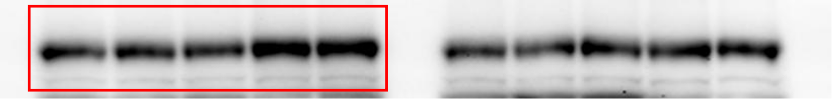

Fig6J-p-AKT

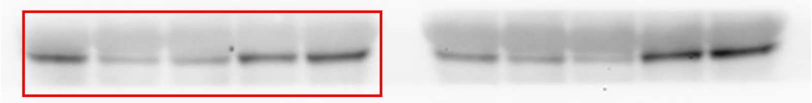

Fig6J-AKT

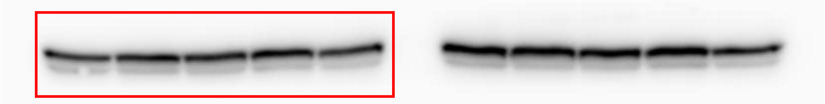

Fig6J-NQO1

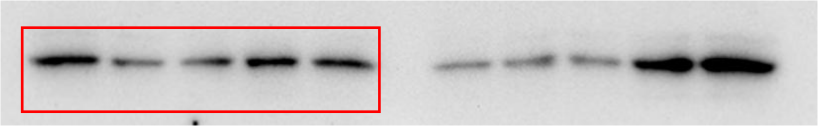

Fig6J-GAPDH

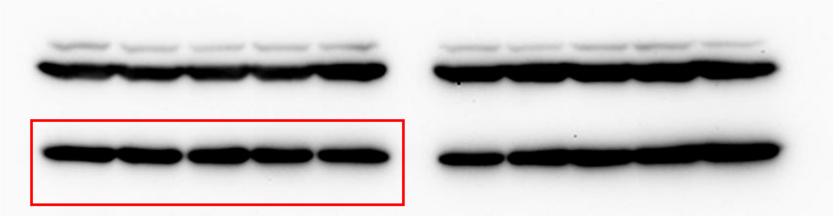

Fig7J-MED23

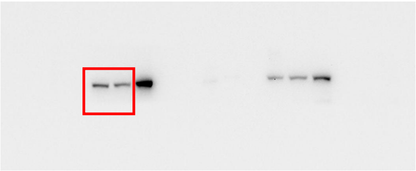

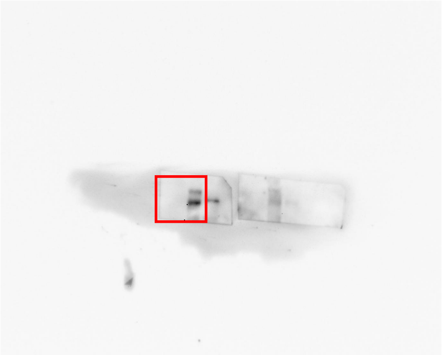

Fig7J-FLAG

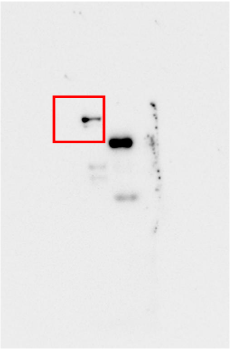

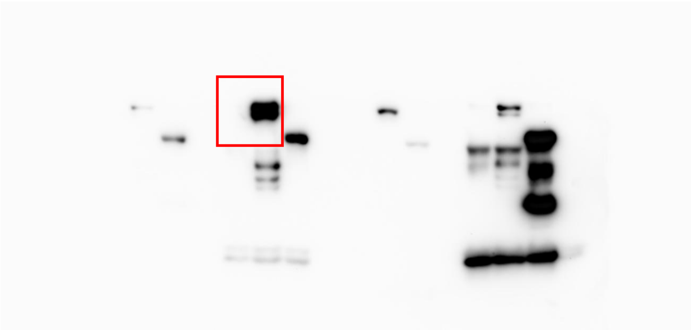

Fig7K-MED23

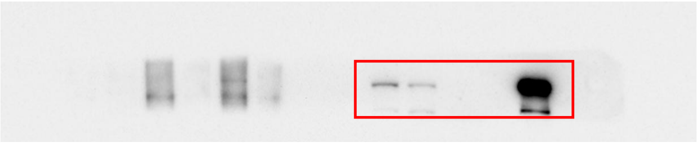

Fig7K-RFX5

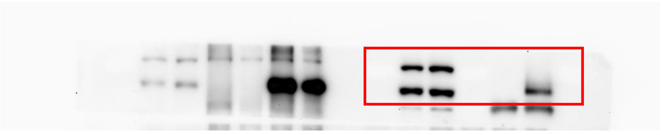

Fig7K-MED6

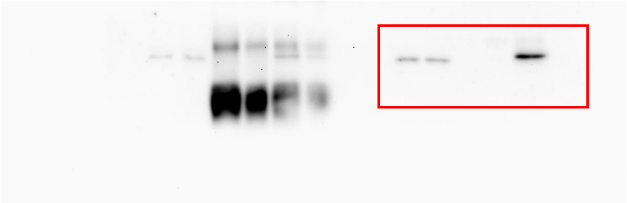

FigS2B-MED23

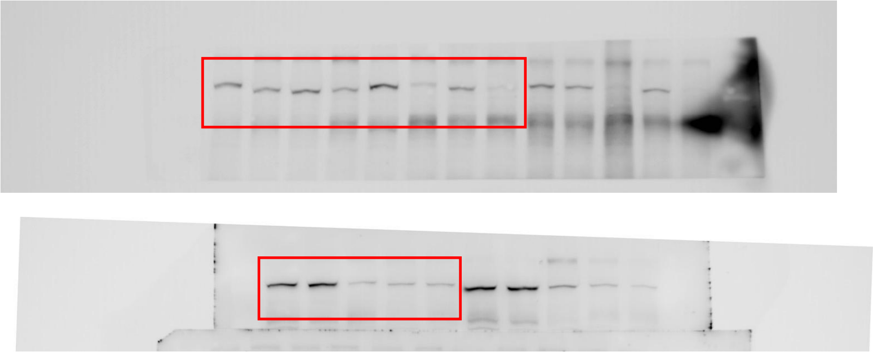

FigS2B-γ-TUBULIN-β-ACTIN-GAPDH

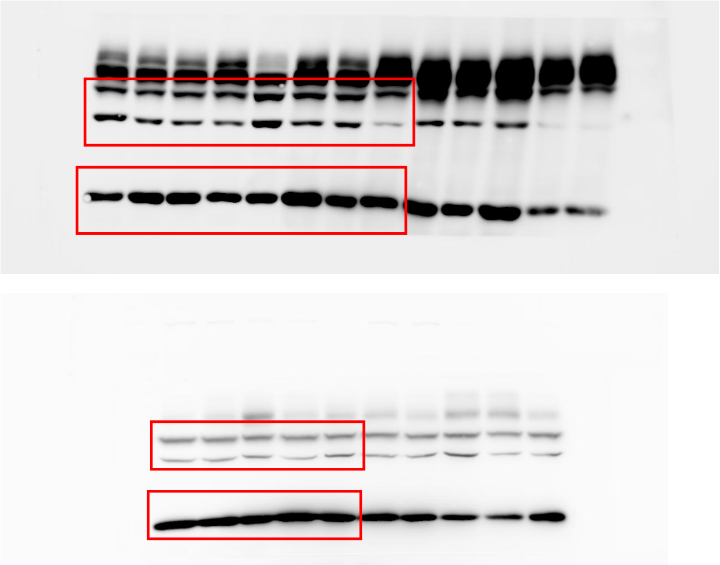

FigS3A-MED23

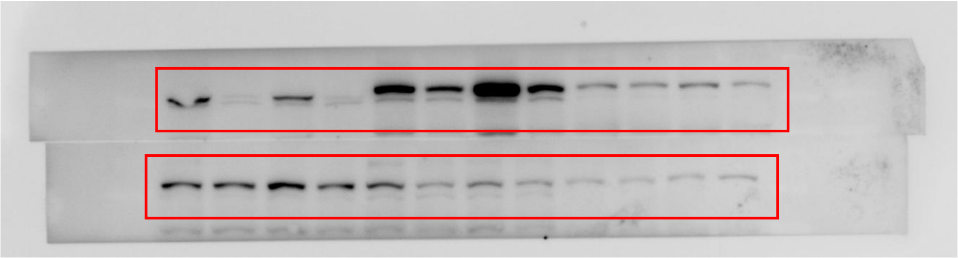

FigS3A-γ-TUBULIN-β-ACTIN

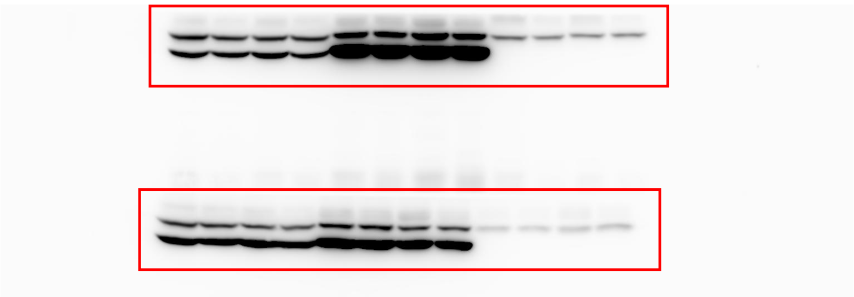

FigS3A-GAPDH

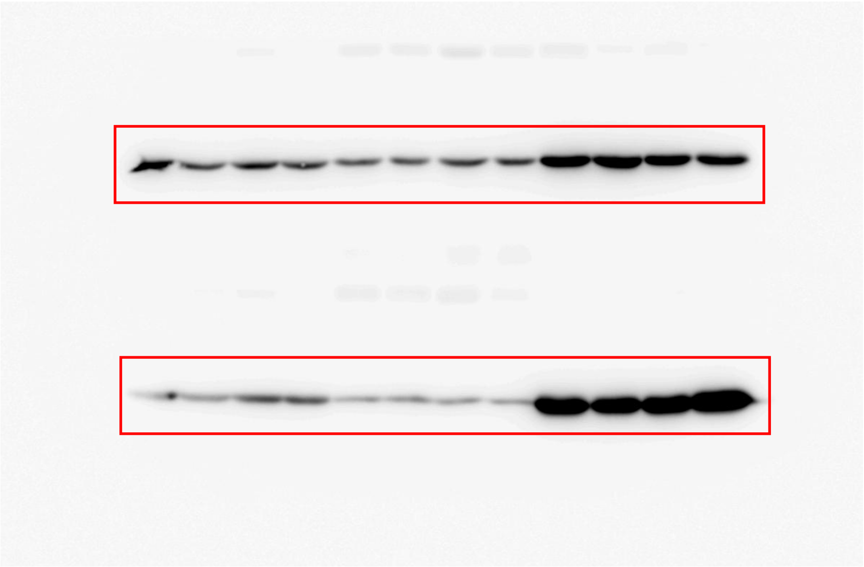

FigS5D-MED23

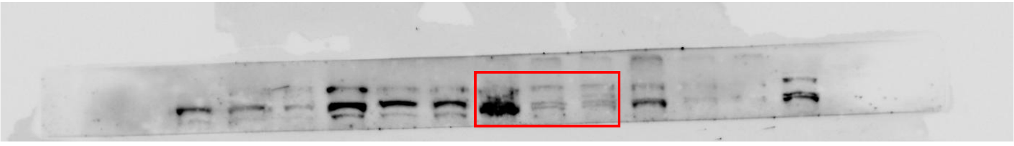

FigS5D-NQO1

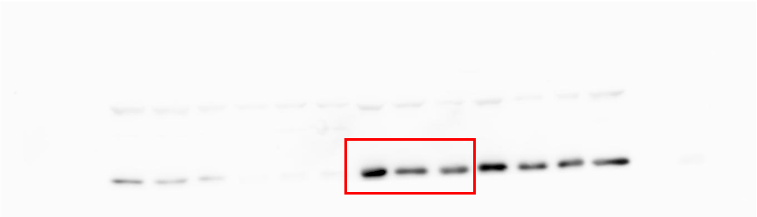

FigS5D-β-ACTIN

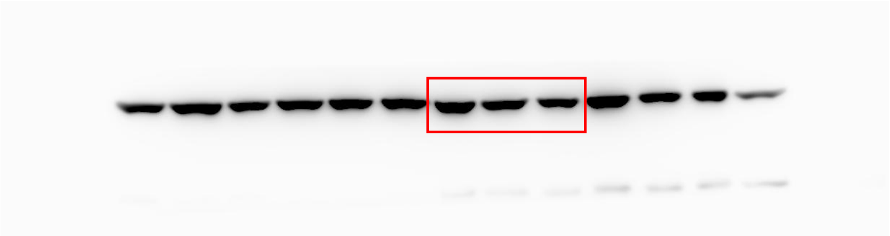

FigS6F-p-IGF1R

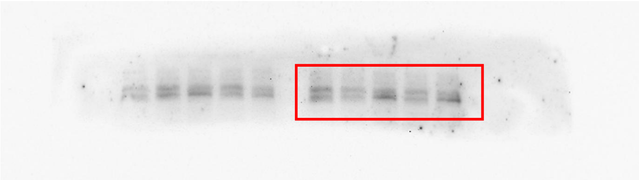

FigS6F-IGF1R

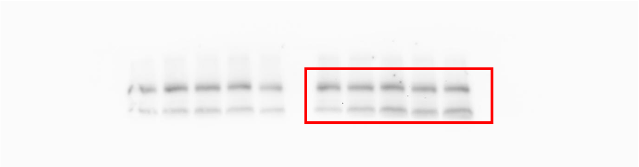

FigS6F-p-AKT

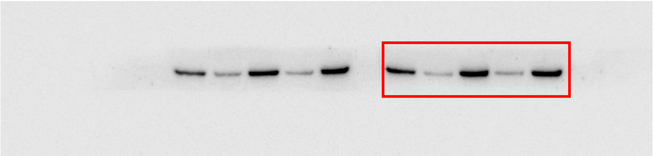

FigS6F-AKT

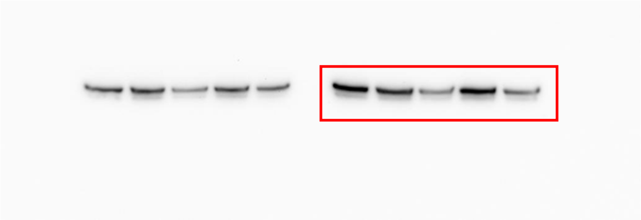

FigS6F-NQO1

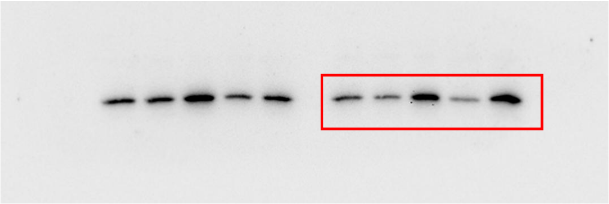

FigS6F-GAPDH

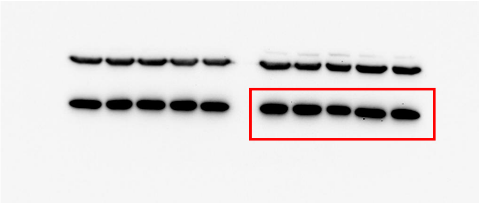

FigS7E-RFX5

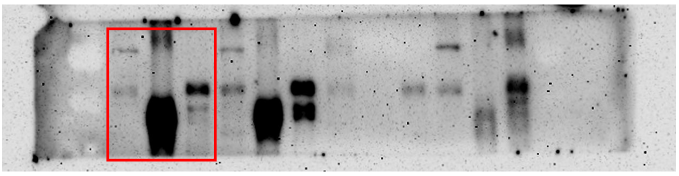

FigS7E-MED6

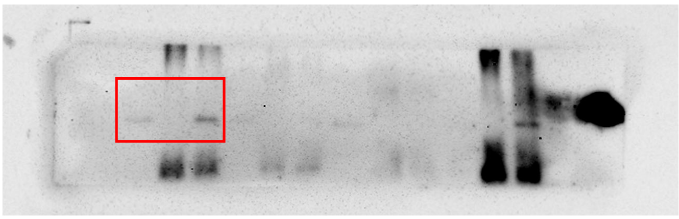

FigS7E-MED23

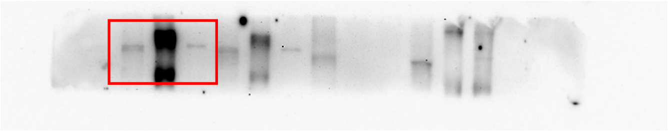

Supplement: Supplementary file 11 — western [file 41419_2025_8348_MOESM11_ESM.pdf]
